# Distinct intestinal microbial signatures linked to accelerated systemic and intestinal biological aging

**DOI:** 10.1186/s40168-024-01758-4

**Published:** 2024-02-22

**Authors:** Shalini Singh, Leila B. Giron, Maliha W. Shaikh, Shivanjali Shankaran, Phillip A. Engen, Zlata R. Bogin, Simona A. Bambi, Aaron R. Goldman, Joao L. L. C. Azevedo, Lorena Orgaz, Nuria de Pedro, Patricia González, Martin Giera, Aswin Verhoeven, Elena Sánchez-López, Ivona Pandrea, Toshitha Kannan, Ceylan E. Tanes, Kyle Bittinger, Alan L. Landay, Michael J. Corley, Ali Keshavarzian, Mohamed Abdel-Mohsen

**Affiliations:** 1 Vaccine and Immunotherapy Center, The Wistar Institute, 3601 Spruce Street, Philadelphia, PA 19104 USA; 2 Rush Center for Integrated Microbiome and Chronobiology Research, Rush University, Chicago, IL USA; 3 Department of Medicine, Rush University, Chicago, IL USA; 4 Life Length, Madrid, Spain; 5 Center for Proteomics and Metabolomics, Leiden University Medical Center, Leiden, The Netherlands; 6 University of Pittsburgh, Pittsburgh, PA USA; 7 Division of Gastroenterology, Hepatology, and Nutrition, Children’s Hospital of Philadelphia, Philadelphia, PA USA; 8 Weill Cornell Medicine, New York, NY USA

**Keywords:** HIV, Biological aging, Aging clocks, Microbiome, Metabolome, Intestines, Gut

## Abstract

**Background:**

People living with HIV (PLWH), even when viral replication is controlled through antiretroviral therapy (ART), experience persistent inflammation. This inflammation is partly attributed to intestinal microbial dysbiosis and translocation, which may lead to non-AIDS-related aging-associated comorbidities. The extent to which living with HIV — influenced by the infection itself, ART usage, sexual orientation, or other associated factors — affects the biological age of the intestines is unclear. Furthermore, the role of microbial dysbiosis and translocation in the biological aging of PLWH remains to be elucidated. To investigate these uncertainties, we used a systems biology approach, analyzing colon and ileal biopsies, blood samples, and stool specimens from PLWH on ART and people living without HIV (PLWoH) as controls.

**Results:**

PLWH exhibit accelerated biological aging in the colon, ileum, and blood, as measured by various epigenetic aging clocks, compared to PLWoH. Investigating the relationship between microbial translocation and biological aging, PLWH had decreased levels of tight junction proteins in the intestines, along with increased microbial translocation. This intestinal permeability correlated with faster biological aging and increased inflammation. When investigating the relationship between microbial dysbiosis and biological aging, the intestines of PLWH had higher abundance of specific pro-inflammatory bacteria, such as *Catenibacterium* and *Prevotella*. These bacteria correlated with accelerated biological aging. Conversely, the intestines of PLWH had lower abundance of bacteria known for producing the anti-inflammatory short-chain fatty acids, such as *Subdoligranulum* and *Erysipelotrichaceae*, and these bacteria were associated with slower biological aging. Correlation networks revealed significant links between specific microbial genera in the colon and ileum (but not in feces), increased aging, a rise in pro-inflammatory microbe-related metabolites (e.g., those in the tryptophan metabolism pathway), and a decrease in anti-inflammatory metabolites like hippuric acid.

**Conclusions:**

We identified specific microbial compositions and microbiota-related metabolic pathways that are intertwined with intestinal and systemic biological aging. This microbial signature of biological aging is likely reflecting various factors including the HIV infection itself, ART usage, sexual orientation, and other aspects associated with living with HIV. A deeper understanding of the mechanisms underlying these connections could offer potential strategies to mitigate accelerated aging and its associated health complications.

Video Abstract

**Supplementary Information:**

The online version contains supplementary material available at 10.1186/s40168-024-01758-4.

## Introduction

The gastrointestinal (GI) tract plays a crucial role in the pathogenesis and persistence of HIV infection [1]. HIV infection disrupts the intestinal epithelial barrier [2–4], leading to increased gut permeability to microbial organisms and their products [5]. This heightened permeability could contribute to local and systemic inflammation, further stimulating HIV replication [5–10] and contributing to the development of non-AIDS-related aging-associated comorbidities [5, 11–18]. This inflammation may also play a role in sustaining viral persistence [7, 8, 10]. Unfortunately, even with the use of antiretroviral therapy (ART), the damage to the gut's epithelial barrier caused by HIV remains unrepaired, allowing microbial translocation and inflammation to persist [19–21].

Several studies have indicated that HIV-related microbial translocation may be exacerbated by changes in the composition and diversity of the gut microbiota, known as microbial dysbiosis. This dysbiosis involves the depletion of putative anti-inflammatory, beneficial bacteria and/or the accumulation of putative pro-inflammatory, harmful bacteria. Among the beneficial bacteria are those that produce short-chain fatty acids (SCFAs) – key metabolites that fortify the intestinal barrier [22–28]. However, it remains unclear whether HIV itself or other factors associated with living with HIV, such as ART usage and sexual orientation, cause this microbial dysbiosis. In people living with HIV (PLWH), some dysbiosis can be attributed to behaviors like sexual activity [29]. Recent findings, however, show that HIV-associated dysbiosis can occur independently of sexual orientation [30]. Likely, the HIV-associated dysbiosis is multifactorial in nature, caused in part by the HIV infection itself, sexual orientation, and additional factors associated with living with HIV. Nevertheless, this living-with-HIV-associated microbial dysbiosis is linked to greater systemic inflammation, exacerbated disease progression, and an increase in inflammation and aging-related conditions [30].

The increased occurrence of inflammation- and age-associated comorbidities in PLWH on ART has prompted recent investigations into whether living with HIV — influenced by the infection itself, ART usage, sexual orientation, or other associated factors — accelerates the process of biological aging. Recent reports have indeed shown evidence of accelerated or heightened biological aging in the blood of PLWH [31–36]. However, these studies have focused on blood cells, despite the potentially important role of the intestines in regulating chronic inflammation in PLWH. Thus, it remains unclear whether living with HIV, in combination with its associated factors, affects the aging process in the intestines. Additionally, it is unknown whether microbial dysbiosis and translocation contribute to the systemic acceleration of biological aging in PLWH on ART. This study aims to address these two knowledge gaps.

## Methods

### Study cohort

Ileum and colon biopsies, blood, and stool samples were collected from 25 PLWH on suppressive ART and 23 people living without HIV (PLWoH) with similar age-, sex-, BMI-, and ethnicity (Table 1) at Rush University Medical Centre. All participants provided informed written consent. Each participant filled out a detailed questionnaire about their demographics, medical history, and ART regimen. Additional details about the study cohort are available in Table 1. We did not include samples from individuals who adhered to special diets, including vegan, vegetarian, gluten-free, Paleo, or specific carbohydrate diets because these diets can impact the microbial community. We also did not include samples from individuals if they had Celiac disease. The study protocol was approved by the Institutional Review Board at Rush University (ORA# 19020710).

**Table 1:** Demographic and clinical characteristics of study participants

		**PLWoH (*n*=23)**	**PLWH on ART (*n*=25)**	***P* value**
**Race; n (%)**^**a**^	White	12 (52%)	13 (52%)	0.3880
	Black	8 (35%)	9 (36%)	
	Other	1 (4%)	3 (12%)	
	White/Asian	2 (9%)	0 (0%)	
**Ethnicity; n (%)**^**b**^	Hispanic	3 (13%)	3 (12%)	>0.99
	Non Hispanic	20 (87%)	22 (88%)	
**Gender; n (%)**^**b**^	Male	18 (78%)	21 (84%)	0.7195
	Female	5 (22%)	4 (16%)	
**Sexual orinetation; n(%)**^**a**^	Heterosexual	21 (91.3%)	7 (28%)	<0.0001
	Homosexual/MSM	0 (0%)	18 (72%)	
	Unknown	2 (8.3%)	0 (0%)	
**Age; median (IQR)**^**c**^		51 (11)	54 (13)	0.5901
**BMI ; median (IQR)**^**c**^		28.01 (4.54)	28.47 (10.545)	0.3811
**BMI category**^**a**^	Normal Weight (<25), %	6 (26%)	6 (24%)	0.3026
	Overweight (25-30), %	12 (52%)	8 (32%)	
	Obese (30-35), %	3 (13%)	4 (16%)	
	Morbidly Obese (>35), %	2 (9%)	7 (28%)	
**CD4 count (cells/mm**^**3**^**); median (IQR)**		-	593 (395)	
**Nadir CD4 count (cells/mm**^**3**^**); median (IQR)**		-	216 (227)	
**HIV load (copies/ml plasma); n (%)**	Not detected	-	2 (8%)	
	<20	-	15 (60%)	
	<40	-	7 (28%)	
	41	-	1 (4%)	
**ART Regimen; n (%)**	Elvitegravir (INSTI), Cobicistat, Emtricitabine (NRTI), Tenofovir alafenamide (NRTI)	-	2 (8%)	
	Bictegravir (INSTI), Emtricitabine (NRTI), Tenofovir alafenamide (NRTI)	-	11 (44%)	
	Abacavir (NRTI), Dolutegravir (INSTI), Lamivudine (NRTI)	-	3 (12%)	
	Emtricitabine (NRTI), Rilpivirine (NNRTI), Tenofovir alafenamide (NRTI)	-	4 (16%)	
	Dolutegravir (INSTI), Lamivudine (NRTI)	-	2 (8%)	
	Dolutegravir (INSTI), Doravirine (NNRTI)	-	1 (4%)	
	Dolutegravir (INSTI), Darunavir (PI), Cobicistat	-	1 (4%)	
	Dolutegravir (INSTI), Emtricitabine (NRTI), Tenofovir alafenamide (NRTI), Doravirine (NNRTI)	-	1 (4%)	
**Diabetes status; n (%)**^**b**^	No	23 (100%)	21 (84%)	0.1105
	Yes	0 (0%)	4 (16%)	
**Liver enzymes; median (IQR)**^**c**^	AST (U/L)	19 (6)	24 (14.5)	0.0343
	ALT (U/L)	20 (10)	23 (20.5)	0.0800
**Hepatitis status; n (%)**^**b**^	No	22 (96%)	17 (68%)	0.0045
	Yes	0 (0%)	8 (32%)	
	Sub-Category :			
	Hx Hep C	0 (0%)	3 (12%)	
	Hx Hep B	0 (0%)	2 (8%)	
	Hx Hep A w/ coma	0 (0%)	1 (4%)	
	Others (e.g., fatty liver, alcoholic steatohepatitis	0 (0%)	2 (8%)	
	N/A	1 (4%)	0 (0%)	
**Neurological diseases status; n (%)**^**b**^	No	20 (87%)	13 (52%)	0.0129
	Yes	3 (13%)	12 (48%)	
**Gut diseases status; n (%)**^**b**^	No	18 (78%)	13 (52%)	0.1246
	Yes	5 (22%)	11 (44%)	
	Sub-Category :			
	GERD	5 (22%)	8 (32%)	
	Crohn's	0 (0%)	1 (4%)	
	Hx Candida esophagitis	0 (0%)	1 (4%)	
	Other (e.g., gastrectomy)	0 (0%)	1 (4%)	
	N/A	0 (0%)	1 (4%)	
**Heart diseases status; n (%)**^**b**^	No	16 (70%)	6 (24%)	0.0033
	Yes	7 (30%)	19 (76%)	
	Sub-Category :			
	Hypertension	2 (21.7%)	10 (40%)	
	Others (e.g., Tachycardia, High Blood pressure)	5 (8.7%)	9 (36%)	

^a^Chi-sq test

^b^Fisher's exact Test

^c^Mann-Whitney U Test

### DNA and RNA isolation

DNA and RNA from colon and ileum biopsies (stored in RNAlater solution by Thermo Fisher) were isolated using the AllPrep DNA/RNA/Protein mini kit (Qiagen, catalog #80004). Briefly, the biopsies were homogenized in RLT Lysis buffer (Allprep isolation kit, Qiagen, catalog #80204) using stainless-steel beads (5 mm, Qiagen, Catalog #69989) on a Qiagen TissueLyser II. Total RNA and DNA were simultaneously purified from the tissue lysates following the manufacturer's protocol. On-column DNAse digestion was performed during the RNA extraction. Quantification of DNA and RNA was performed using a Nanodrop (ND-1000) spectrophotometer.

### Quantification of DNA methylation and epigenetic clock calculations

Three hundred ng of DNA per sample was bisulfite-converted using the EZ DNA Methylation kit (Zymo Research) following the manufacturer’s instructions. These bisulfite-converted DNA samples were randomly assigned to a chip well on the Infinium HumanMethylationEPIC v1.0 BeadChip, amplified, hybridized onto the array, stained, washed, and imaged with the Illumina iScan SQ instrument to obtain raw image intensities. Raw Methylation EPIC array IDAT intensity data were loaded and preprocessed in the R statistical programming language (http://www.r-project.org) using the SeSAMe analysis suite R package [37]*.* Epigenetic estimates for Horvath’s multi-tissue predictor DNAmAge based on 353 CpG sites [38], the Horvath skin-and-blood clock based on 391 CpG sites [39], Levine DNAmPhenoAge based on 513 CpG sites [40], Hannum’s clock based on 71 CpG sites [41], the Lu’s telomere length predictor [42], and DNA methylation-based mortality risk assessment (GrimAge [43]) were calculated. Principal component-based epigenetic clock estimates were calculated utilizing an R script provided by Higgin-Chen et al. and 78,464 CpGs for each sample in a beta matrix [44]. Mean imputation was utilized for missing values. DunedinPACE pace of aging was calculated using the publicly available Github code [45]*.*

### Telomere length quantification

Telomere length quantification in peripheral blood mononuclear cells (PBMCs) was performed using a high-throughput (HT) Q-FISH (quantitative fluorescent in situ hybridization) method developed by Life Length Technologies [46]. Briefly, the cells were fixed and hybridized with a fluorescent Peptide Nucleic Acid (PNA) probe that recognizes three telomere repeats. After hybridization, the cells were thoroughly washed to remove non-specific binding, and the nuclei were stained with DAPI. The images of the nuclei and telomeres are captured by a high-content screen system (Opera Phenix, Perkin Elmer) using maximum projection image from several Z-stack individual images, to get a more reliable image of the telomere. The fluorescent intensities were translated to base pair through a standard regression curve which is generated using control cell lines with known telomere lengths. Data were analyzed using proprietary software (TAT Analyzer) to generate all telomere-associated variables (TAVs). The data generated a TAV profile with descriptive statistics of telomere length, values for each percentile of telomere length, percentages of telomere length values, percentages of cells with specific telomere values, and dispersion parameters for each sample.

### Quantification of cell-associated HIV-1 DNA and RNA

Total cell-associated HIV DNA and RNA were quantified using a qPCR TaqMan assay with specific primers to amplify the LTR: F522-43 (5’ GCC TCA ATA AAG CTT GCC TTG A 3’; HXB2522–543) and R626-43 (5’ GGG CGC CAC TGC TAG AGA 3’; 626–643), coupled with a FAM-BQ probe (5’ CCA GAG TCA CAC AAC AGA CGG GCA CA 3), on a QuantStudio 6 Flex Real-Time PCR System (Applied Biosystems). Cell counts were normalized by qPCR using human genomic TERT (Telomerase Reverse Transcriptase) for DNA and RPLP0 expression for RNA (Life Technologies), respectively. For determination of cell-associated HIV DNA copy number, a 20 μl PCR reaction containing 10 μl of 2 × TaqMan Universal Master Mix II including UNG (Life Technologies), 4 pmol of each primer, 4 pmol of probe, and 5 μl of DNA was prepared. The following cycling conditions were used: 50 °C for 2 min, 95 °C for 10 min, followed by 60 cycles of 95 °C for 15 s and 59 °C for 1 min. For determination of cell-associated HIV RNA copy number, a 20 μl PCR reaction containing 10 μl of 2 × TaqMan RNA to Ct 1 Step kit (Life Technologies), 4 pmol of each primer, 4 pmol of probe, 0.5 μl reverse transcriptase, and 5 μl of RNA was used. Cycling conditions were 48 °C for 20 min, 95 °C for 10 min, followed by 60 cycles of 95 °C for 15 s and 59 °C for 1 min. For absolute HIV DNA measurement, external quantitation standards were prepared by DNA extraction from ACH-2 cells; and calibrated to the Virology Quality Assurance (VQA, NIH Division of AIDS) cellular DNA quantitation standards. For HIV RNA measurements, external quantitation standards were prepared from full-length NL4-3 virion RNA, and copy numbers were determined using the Abbott RealTime assay (Abbott Diagnostics, Des Plains, Ill), calibrated to VQA HIV-1 RNA standards. Up to 500 ng of total cellular RNA or DNA was added to each reaction well. HIV RNA or DNA copy numbers were determined by extrapolation against a 7-point standard curve (1–10,000 cps) performed in triplicate.

### Assessing tight junction proteins in colon and ileum

Ileal and colonic biopsies were embedded in optimal cutting temperature (OCT) and cut into 5-μm thick sections. Subsequently, they were fixed in a 1:1 acetone/methanol solution at -20ºC for 2 min. The slides were air-dried and then rinsed in 1X PBS for 10 min for rehydration. Afterward, the slides were permeabilized at 40ºC for 5 min in a 0.2% Triton X-100/PBS solution, washed and blocked for 1 h at room temperature using a 2% non-fat dry milk solution. Following this, the slides were stained with primary antibodies against ZO-1 (Invitrogen, catalog# 61–7300) or occludin (Invitrogen, catalog # 33–1500) diluted in 2% milk for 1 h at 37ºC, washed; and stained with secondary antibodies (Invitrogen Alexa Fluor donkey anti-rabbit 488 #A-21206 or Alexa Fluor donkey anti-mouse 488 #A-21202) at a dilution of 1:250 for 1 h at 37ºC. Subsequently, the slides were washed and nuclei were stained with DAPI for 3 min, washed, and mounted using Fluoromount™ Aqueous Mounting Medium (Sigma, Catalog #F4680). Images from at least five stained tissue fields per sample were used to determine the relative expression of each marker and select representative images. All staining images were evaluated by two independent, blinded observers using a Zeiss Axio Observer 7 digital deconvolution immunofluorescent microscope (Zeiss, Oberkochen). All images were captured at 40 × magnification.

### 16S rRNA gene library preparation

DNA was extracted from approximately 200 mg of stool or tissue using the Qiagen DNeasy PowerSoil Pro kit and quantified using the Quant-iT PicoGreen Assay Kit. Barcoded PCR primers targeting the V1-V2 region of the 16S rRNA gene were used for library generation. PCR reactions were performed in duplicate using Q5 High-Fidelity DNA Polymerase (NEB). For high microbial biomass samples such as fecal material, each 50 µl PCR reaction mix contained 0.5 µM of each primer, 0.34 U Q5 Pol, 1X Buffer, 0.2 mM dNTPs, and 5 µl DNA. Cycling conditions were as follows: 1 cycle of 98 °C for 1 min; 20 cycles of 98 °C for 10 s, 56 °C for 20 s, and 72 °C for 20 s; 1 cycle of 72 °C for 8 min. The PCR reactions for low microbial biomass samples such as colon and ileum tissues, were performed similarly, with 10 µl DNA. Cycling conditions were as follows: 1 cycle of 98 °C for 1 min; 25 cycles of 98 °C for 10 s, 56 °C for 20 s, and 72 °C for 20 s; 1 cycle of 72 °C for 8 min. Samples amplified in duplicates, were pooled, and purified using a 1:1 volume of SPRI beads. DNA in each sample was then quantified using PicoGreen and pooled in equal molar amounts. The resulting library was sequenced on the Illumina MiSeq using 2 × 250 bp chemistry. Extraction blanks and DNA-free water subjected to the same amplification and purification procedure were used to allow for empirical assessment of environmental and reagent contamination. Positive controls, consisting of eight artificial 16S gene fragments synthesized in gene blocks and combined in known abundances, were also included. No contamination was observed in the negative controls, and the positive control samples produced the expected read counts (Supplementary Table 1). We acquired an average of 81.4 k raw reads and 51.9 k quality control (QC)-filtered reads per sample (Supplementary Table 1). Five samples were excluded from the final analysis due to insufficient (< 1000) QC-filtered reads, and the average read counts of the samples used in the final analysis were 84.6 k raw reads and 53.5 k QC-filtered reads per sample, with a minimum of 5 k raw reads and 3.9 k QC-filtered reads (Supplementary Table 1).

### 16S rRNA gene bioinformatics processing

16S rRNA sequence data were processed using QIIME2 version 2019.7 [47]. Read pairs were processed to identify amplicon sequence variants with DADA2 truncating both forward and reverse reads at 240 nucleotides [48]. Taxonomic assignments were generated by comparison to the SILVA reference database version 132 [49], using the naive Bayes classifier implemented in scikit-bio using default parameters [50]. Numbered taxa, such as *Prevotella* 2, represent distinct taxonomic groups in the SILVA taxonomy. Data files from QIIME were analyzed in the R environment for statistical computing [51]. Linear mixed-effects models were used to estimate the mean difference between sample types. Linear models were used to estimate the mean difference between controls and PLWH on ART group for each sample type. Relative abundances were log10-transformed for the tests. Only the bacteria with at least 1% mean relative abundance in at least one sample type were tested. Correlations of interest between markers and bacterial abundances were determined using Spearman correlation. When multiple tests were performed, p-values were corrected for false discovery rate using the Benjamini–Hochberg method [52]. An inferred modelling approach, using 16S rRNA microbial relative abundances of the genus taxonomic level, allowed us to identify individual taxa and group them accordingly based on their known involvement with total SCFA-production, total butyrate-production, and putative proinflammatory-production. Total butyrate-producing bacteria were identified from extensive research literature searches on PubMed [53–59]. Many gram-positive anti-inflammatory beneficial butyrate-producing genera identified in this analysis belong to the clostridial cluster XIVa and IV (family Lachnospiraceae and Ruminococcaceae respectively) within the phyla Firmicutes. Assessment of these predictive microbial percent relative abundance ratios were calculated and compared between PLWH and controls at all three sample sites [60, 61].

### Untargeted measurement of stool metabolites

About 200 mg of stool, per sample, were dissolved in 250µL of LC–MS quality water containing 0.2 mM NaN_3_. The samples were subjected to bead beating for 30 s using 0.5 mm zirconium oxide ceramic beads in a Bullet Blender 24 (Next Advance Inc.). 750µL of methanol was added to each tube and samples were centrifuged at 16,000 xg at 4˚C for 15 min. 800 µL of the supernatant were transferred to new tubes and dried in a Speedvac (Eppendorf, model 5301). Dried samples were reconstituted in 200µL of a 1% methanol in water solution and centrifuged at 16,000 xg at 4 °C for 5 min. Samples were further diluted by tenfold in 1% methanol in water solution, and transferred to micro-vial inserts and placed in the autosampler. A QC sample was made by pooling 10 µL of each sample and was analyzed periodically across the metabolomics run. Metabolomic analysis of the fecal extracts were performed using a liquid chromatography tandem mass spectrometry (LC–MS/MS) system using an in-house metabolite library. A Shimadzu Nexera X2 (consisting of two LC30AD pumps, a SIL30AC autosampler, a CTO20AC column oven and a CBM20A controller; Shimadzu, The Netherlands) was used to deliver a programmed gradient of water (eluent A) and methanol (eluent B), both containing 0.1% formic acid. The gradient, using a flow of 0.4 mL/min, was 0% B at 0 min, 0% B at 1.5 min, 97% B at 9.9 min, 97% B at 12.9 min, 0% B at 13.0 min and 0% B at 13.8 min. A Synergi Hydro-RP, 2.5 µm particles, 100 × 2 mm was used as column with a Phenomenex SecurityGuard Ultra C8, 2.7 µm, 5 × 2.1 mm cartridge as guard column. The column was kept at 40˚C and the injection volume was 2 µL. The MS was a Sciex TripleTOF 6600 (AB Sciex Netherlands B.V., The Netherlands) operated in positive and negative ESI mode, with the following conditions: ion source gas 1 50 psi, ion source gas 2 50 psi, curtain gas 30 psi, temperature 500 °C, acquisition range *m/z* 75–650, ion spray voltage 5500 V (ESI +) and -4500 V (ESI-), and declustering potential 80 V (ESI +) and -80 V (ESI-). An information dependent acquisition (IDA) method was used to identify the different metabolites, with the following conditions for MS analysis: collision energy ± 10, acquisition time 80 ms and for MS/MS analysis: collision energy ± 30, collision energy spread 15, ion release delay 30, ion release width 15 and acquisition time 40 ms. The IDA switching criteria was to exclude isotopes within 4 Da for a maximum of 18 candidate ions to monitor per cycle. MS-DIAL (v4.90) [62], with our in-house metabolite database was used to align the data and identify the different metabolites matching accurate mass, retention time and in most cases, the MS/MS fragmentation pattern against the authentic chemical standards. Metabolites with the peak area’s RSD below 30% in the QC samples and with sample-to-blank ratio above 5 for at least 80% of the samples within the experimental groups were considered for further data analysis. Normalization was done using the stool sample weight.

### Measurement of plasma inflammatory markers

Plasma levels of fractalkine, IFN-α2a, IL-12p70, IL-2, IL-4, IL-5, IP-10, MCP-2, MIP-1α, SDF-1α, eotaxin, IFN-β, IFN-γ, IL-10, IL-1β, IL-21, IL-6, Leptin, CXCL9, and TNF-α were determined using U-PLEX kits from Meso Scale Diagnostics (Biomarker Group 1 (hu) Assays; catalog # K151AEM-2, Custom Immuno-Oncology Grp 1 (hu) Assays; catalog # K15067L-2, and Human MIG (CXCL9) Antibody Set; catalog # F210I-3) according to the manufacturer's instructions. Levels of Growth Differentiation Factor-15 (GDF-15) were measured by ELISA using the Human GDF-15 DuoSet ELISA Kit (R&D Systems; catalog # DY957). Plasma levels of Myeloperoxidase (MPO), and C3a were measured by ELISA (Thermo Fischer; catalog # BMS2038INST, and #BMS2089).

### Measurement of plasma markers of tight junction permeability and microbial translocation

Plasma levels of soluble CD14 (sCD14), soluble CD163 (sCD163), LPS Binding Protein (LBP), and FABP-2/I-FABP were quantified using DuoSet ELISA kits (R&D Systems; catalog # DY383-05, # DY1607-05, # DY870-05, and # DFBP20 respectively). The plasma level of Zonulin was measured using an ELISA kit from MyBiosorce (catalog # MBS167049). β-D-glucan detection in plasma was performed using Limulus Amebocyte Lysate (LAL) assay (Glucatell Kit, CapeCod; catalog # GT003). Levels of occludin were measured by ELISA (Biomatik; catalog # EKC34871), and levels of Reg3A were measured by ELISA (RayBiotech; catalog # ELH-REG3A-1).

### Untargeted measurement of plasma metabolites

Metabolomic analysis was conducted following previously described methods [63, 64]. In brief, polar metabolites were extracted using 80% methanol. A QC sample was created by pooling equal volumes of all samples and injected periodically during the sample sequence. LC–MS was performed on a Thermo Scientific Q Exactive HF-X mass spectrometer with HESI II probe and Vanquish Horizon UHPLC system. Hydrophilic interaction liquid chromatography (HILIC) was performed at 0.2 ml/min on a ZIC-pHILIC column (2.1 mm × 150 mm, EMD Millipore) at 45 °C. All samples were analyzed by full MS with polarity switching, and the QC sample was also analyzed by data-dependent MS/MS with separate runs for positive and negative polarities. Raw data were analyzed using Compound Discover 3.3 SP1 (ThermoFisher Scientific). Accurate mass or retention time was used to identify the metabolites by utilizing an in-house database generated from pure standards or by querying the mzCloud database (www.mzCloud.org) with MS/MS spectral data. Matches with scores of 50 or greater were selected. Metabolite quantification utilized integrated peak areas from full MS runs. These values were corrected based on the periodic QC runs and normalized to the total signal from identified metabolites in each sample.

### Statistical analysis

Statistical analyses were performed using Mann–Whitney U tests, Friedman test, Kruskal–Wallis test, and Spearman's rank correlations. The Benjamini–Hochberg method [52] and the Two-stage step-up method of Benjamini, Krieger, and Yekutieli [65] were used for multiple-comparisons correction. These analyses were conducted using GraphPad Prism release 9.0 (GraphPad Software).

## Results

### PLWH on ART exhibit accelerated biological aging in the intestines, with rates differing from that in the blood

**Fig. 1:**
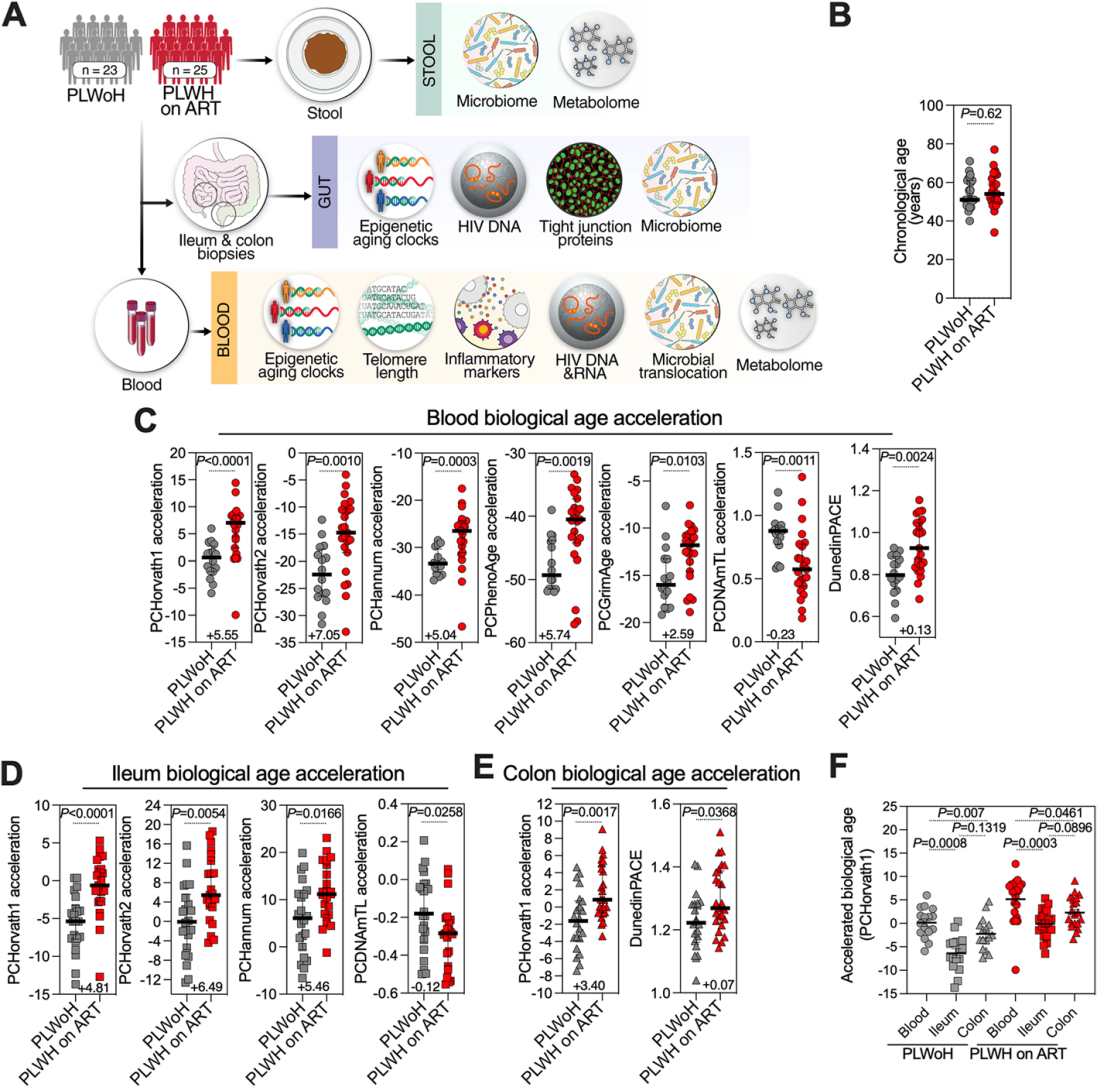
Accelerated intestinal and blood biological aging in PLWH on ART. **A** Study design schematic. **B** Chronological age comparison between PLWoH and PLWH on ART, displaying median and interquartile range (IQR). Statistical analysis was performed using the Mann–Whitney U test. **C-E** Dot-plots illustrating the significant acceleration in biological age for blood (**C**), ileum (**D**), and colon (**E**) based on multiple epigenetic clocks: Horvath1, Horvath2, Hannum, PhenoAge, DNAmTL, GrimAge, and DunedinPACE for blood; Horvath1, Horvath2, Hannum, and DNAmTL for ileum; Horvath1 and DunedinPACE for colon. Positive graph values indicate accelerated biological aging differences per clock, while negative values for DNAmTL signify telomere reduction differences. Both median and IQR are depicted, with statistical analysis via Mann–Whitney U tests. **F** Dot-plots contrasting tissue-specific accelerated biological age differences in blood, ileum, and colon utilizing Horvath1. Statistical assessments were conducted using the non-parametric Friedman test, corrected using the Two-stage step-up method of Benjamini, Krieger, and Yekutieli

We collected colon and ileal biopsies, blood, and stool samples from 25 PLWH on ART with a viral load of < 50 copies/ml and 23 PLWoH matched by age, sex, ethnicity, and BMI (Table 1). Using the systems biology approach illustrated in Fig. 1A, we aimed to determine whether living with ART-suppressed HIV infection is associated with shifts in intestinal biological age, and whether microbial translocation and dysbiosis are linked to biological aging in PLWH on ART.

Patterns of DNA methylation at specific CpG sites have been used to gauge both chronological and biological age across numerous cell types, tissues, and organs in humans and other mammals [38, 40, 41, 43, 66, 67]. Such distinct patterns of DNA methylation form the basis for multiple epigenetic clocks of aging, with different clocks using different sets of CpG sites for their calculations. We first gauged the blood biological age of PLWH on ART and PLWoH using six established DNA methylation epigenetic clocks of aging. Specifically, we used DNA from PBMCs to estimate biological age with the following principal component-based epigenetic clocks [44]: Horvath's multi-tissue predictor DNAmAge (PCHorvath1) based on 353 CpG sites [38], Horvath's skin and blood clock (PCHorvath2) based on 391 CpG sites [66], Hannum's clock based on 71 CpG sites (PCHannum) [41], Levine’s DNAmPhenoAge based on 513 CpG sites (PCPhenoAge) [40], DNA methylation-based mortality risk assessment (PCGrimAge) [43], and Lu’s telomere length predictor (PCDNAmTL) [67]. For five of these clocks, a higher value indicates an older biological age. However, for the PCDNAmTL clock, a lower value indicates older age.

Despite having a similar chronological age (Fig. 1B), the blood of PLWH on ART showed an older biological age than that of PLWoH. The difference ranged from + 3.1 years (using PCGrimAge) to + 7.62 years (using PCHorvath2) (Supplementary Fig. 1A). We next calculated the acceleration of biological age by regressing the outputs of the clocks against chronological age. Larger indices imply faster biological aging, except for PCDNAmTL where a smaller index denotes accelerated aging. This analysis found that the biological age of PLWH on ART was accelerated between 2.59 to 7.05 years (Fig. 1C). We also employed the DunedinPACE epigenetic clock, which estimates the pace of aging [45]. Higher values for this metric correlates with accelerated aging [45]. Consistently, the DunedinPACE estimate was markedly higher in PLWH on ART than in the controls (Fig. 1C), echoing recent studies which suggest that PLWH experience accelerated biological aging in blood [31–36].

Next, we applied the same epigenetic aging clocks to DNA isolated from the ileum and colon (Supplementary Fig. 1B-C). The ileum of PLWH on ART showed accelerated biological aging by four of the seven epigenetic clocks (Horvath1, Horvath2, Hannum, and PCDNAmTL; Fig. 1D), compared to controls. Similarly, the colon of PLWH on ART showed accelerated biological aging using two clocks (Horvath1 and DunedinPACE; Fig. 1E). That some clocks did not detect aging acceleration in the ileum and colon might be because most of these clocks were designed for use on blood samples. Since Horvath1 [38] was developed using tissues, we compared its biological age acceleration across the blood, ileum, and colon samples (Fig. 1F). These data emphasize that the ileum, colon, and blood in PLWH on ART all exhibit accelerated biological aging. However, the acceleration rate differs among tissues.

In our cohort study, there are distinct sexual practices among the two groups of PLWoH and PLWH on ART. Notably, the majority of PLWoH are heterosexual, contrasting with only seven heterosexual individuals in the PLWH on ART group (Table 1). To investigate whether the observed acceleration in biological aging is exclusively linked to sexual practices, we conducted a secondary analysis comparing the rate of acceleration in biological aging specifically among heterosexual participants in both groups. Our findings, detailed in Supplementary Fig. 2, reveal that heterosexual PLWH on ART exhibit a trend towards an increased rate of acceleration in biological aging compared to their heterosexual PLWoH counterparts, despite the small sample size relative to the entire cohort. This suggests a potential contribution of ART-suppressed HIV infection to this accelerated aging phenomenon.

We have also conducted an exploratory analysis to assess how different ART regimens might impact the rate of biological aging acceleration in PLWH on ART. Given the variety of ART regimens in our cohort (Table 1), we categorized the participants into three groups: a) those on bictegravir (Btg) combined with emtricitabine and tenofovir alafenamide (two nucleoside reverse transcriptase inhibitors, NRTIs); b) those on dolutegravir (Dtg) with a varied ART class, including both NRTIs and non-nucleoside reverse transcriptase inhibitors (NNRTIs), alongside protease inhibitors; and c) those not on Btg or Dtg. Notably, each group displayed varying rates of age acceleration compared to PLWoH, particularly in the colon and ileum tissues (Supplementary Fig. 3). Trends indicated a potentially higher rate of biological aging acceleration for PLWH on Dtg-containing ART compared to other groups (Supplementary Fig. 3). Although our sample size restricts us from making conclusions about specific ART regimens associated with higher or lower rates of biological aging, since all participants were on combination ART regimens, these findings suggest a significant influence of ART type on biological aging rates in PLWH. This warrants further research in larger cohorts.

### Epigenetic clock estimates of biological age were validated using other established and emerging markers of aging

**Fig. 2:**
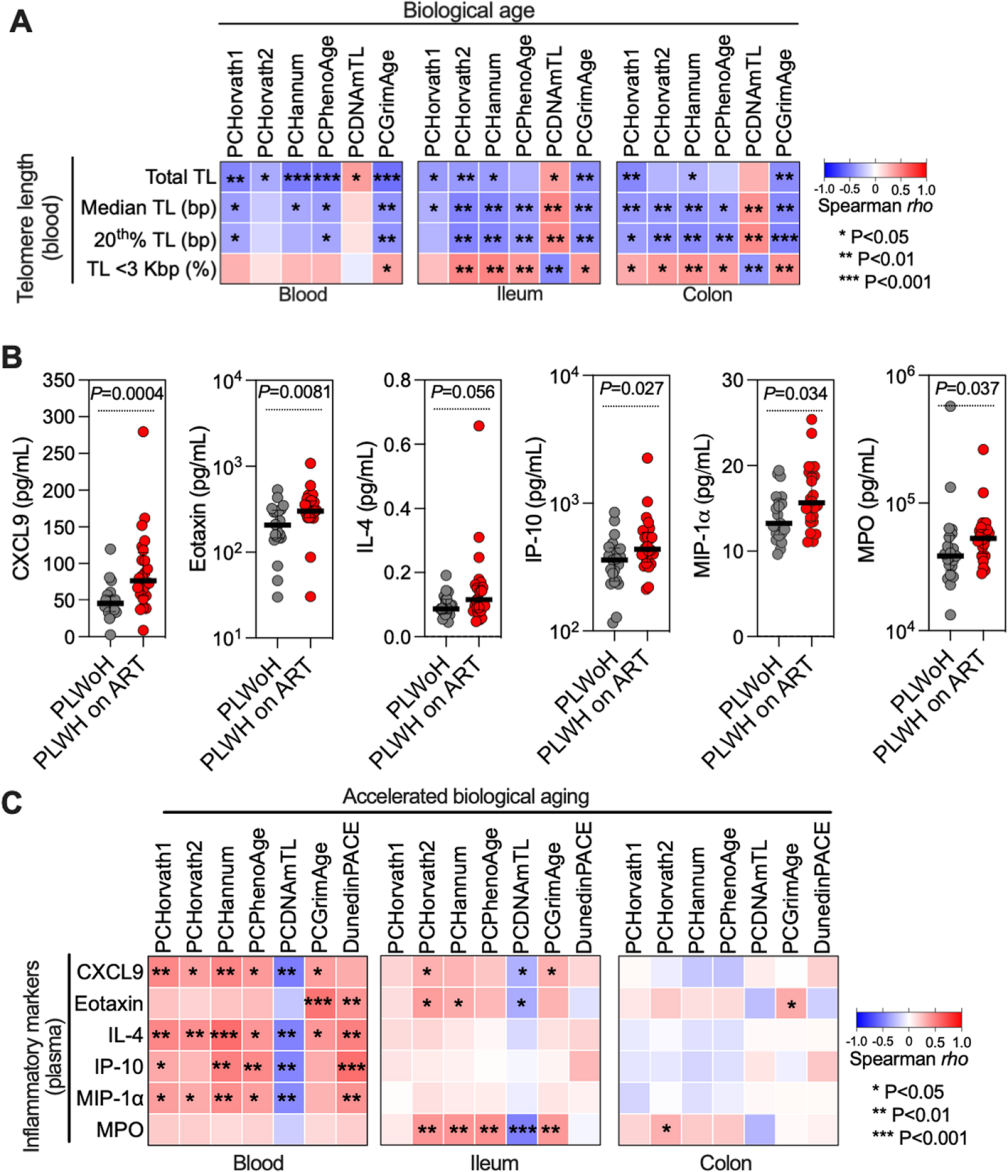
The rate of acceleration in biological age, as assessed by epigenetic clocks, corresponds to other established and emerging methods for measuring biological aging. **A** Spearman's rank correlation heatmap displaying the correlations between telomere lengths in blood (rows) and DNA methylation-based biological aging in blood, ileum, and colon (columns), as gauged by various epigenetic clocks including Horvath1, Horvath2, Hannum, PhenoAge, DNAmTL, and GrimAge. Positive and negative correlations are illustrated in red and blue, respectively.** B** Dot plots depict elevated inflammatory markers in plasma for PLWH on ART compared to PLWoH. Data is represented by medians and interquartile ranges (IQR), with each dot signifying an individual. Comparisons were drawn using the Mann–Whitney U test. **C** Heatmaps of Spearman’s rank correlations between plasma-based inflammatory markers of aging (rows) and accelerated epigenetic age in blood, ileum, and colon (columns), as estimated by the indicated epigenetic clocks. Positive and negative correlations are illustrated in red and blue, respectively. * *P* < 0.05, ** *P* < 0.01, *** *P* < 0.001

To support the results from the epigenetic clocks, we compared these to established and emerging biomarkers of aging. As telomere length (TL) is an established aging marker [68, 69], we evaluated TL in PBMCs via HT-Q-FISH. Median TL did not differ between PLWH on ART and controls (Supplementary Fig. 4A-C); however, PLWH on ART had a higher percentage of cells with shorter telomeres (and a lower percentage with longer telomeres) than controls (Supplementary Fig. 4D). We then determined the correlations between biological age, as estimated by the epigenetic clocks, and measures of TL (Fig. 2A and Supplementary Table 2). These correlations show that higher biological age estimated by the epigenetic clocks correlate strongly with shorter TL.

In addition, new metrics for biological age have recently emerged, including the deep learning based ‘inflammatory aging clock’ called iAge [70]. This metric is derived from the measurement of several inflammation markers in plasma, such as CXCL9 and eotaxin; these are incorporated into an inflammatory aging clock that can predict accelerated aging [70]. We measured the levels of some of the markers included in iAge, as well as other inflammatory indicators pertinent to HIV infection (e.g., IL4, IL-6, MIP-1α) [71, 72] in blood using multiple cytokine arrays. Levels of several markers, including CXCL9 and eotaxin, were elevated in PLWH on ART compared to controls (Fig. 2B). Higher levels of these inflammation markers correlated with accelerated biological aging (derived from the epigenetic clocks as in Fig. 1C-F), especially in blood (Fig. 2C and Supplementary Table 3). Interestingly, the strongest correlation between tissue biological age and markers of inflammation was observed between Myeloperoxidase (MPO) and the biological age of the ileum, but not in the colon. MPO is most abundantly expressed in neutrophils. Neutrophils form clusters in the terminal ileum while being absent from the colon [73]. This might explain the strong association between MPO and aging in the ileum, where neutrophil levels are high, as opposed to the colon, where neutrophil levels are low. We also examined correlations between accelerated aging (based on Horvath1) and the levels of cell-associated HIV DNA in PBMCs, ileum, and colon, and cell-associated HIV RNA in PBMCs, as surrogates for HIV persistence. Among these, the strongest association with epigenetic age acceleration was HIV DNA levels in the ileum (Supplementary Fig. 5). These findings validate the results obtained with the epigenetic clocks and support the conclusion that living with HIV, even with ART, accelerates biological aging in both tissues and blood, with the rate differing among them.

### Intestinal permeability and microbial translocation are linked to accelerated biological aging

**Fig. 3:**
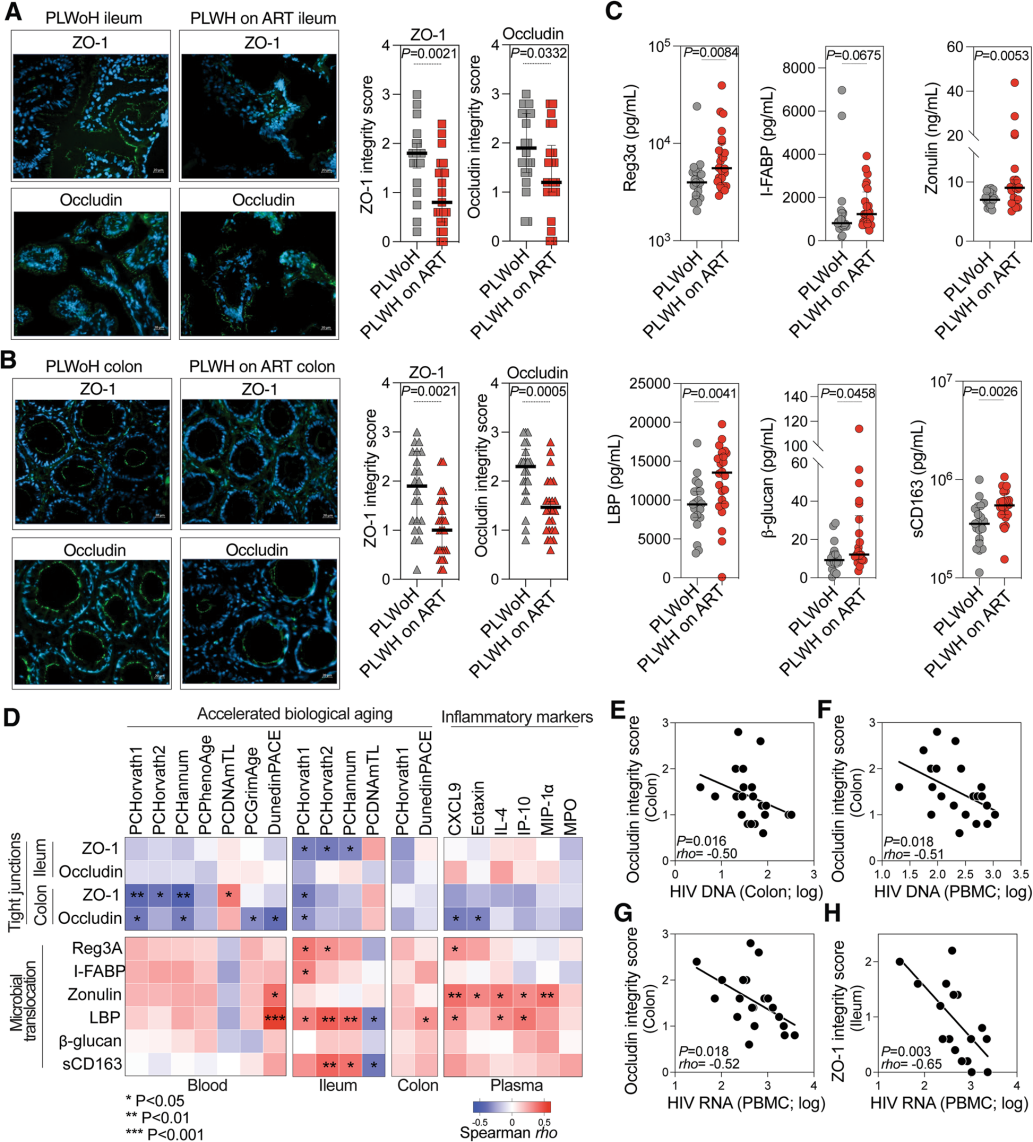
Intestinal permeability and microbial translocation are associated with increased rates of accelerated biological aging. **A-B** Ileum (**A**) and colon (**B**) samples from PLWoH and PLWH on ART depict ZO-1 or occludin expression (green). Nuclei were stained with DAPI (blue). Images, captured at 40 × magnification on a Zeiss Axio Observer 7 microscope, have a scale bar of 20 μm. Tight junction scores are presented as medians with IQR. Statistical significance was determined using Mann–Whitney U tests. **C** Elevated microbial translocation markers in PLWH on ART plasma compared to PLWoH, represented as median with IQR. Statistical significance determined using Mann–Whitney U tests. **D** Heatmaps of Spearman's rank correlations between tight junction integrity (top rows) and microbial translocation (bottom rows) with accelerated biological aging (left columns) and inflammatory markers (right columns). Positive and negative correlations are colored in red and blue, respectively. * *P* < 0.05, ** *P* < 0.01, *** *P* < 0.001. **E–H** Scatter plots display Spearman's rank correlations between HIV DNA/RNA levels in various tissues and tight junction scores: (**E**) HIV DNA in colon vs occludin score in colon, (**F**) HIV DNA in PBMC vs occludin score in colon, (**G**) HIV RNA in PBMC vs occludin score in colon, and (**H**) HIV RNA in PBMC vs ZO-1 score in ileum

Microbial translocation and dysbiosis are increasingly hypothesized to drive systemic inflammation and thus promote inflammation-associated diseases of aging. Given that PLWH on ART experience accelerated biological aging both systemically and within tissues, we explored the possibility that microbial translocation and microbial dysbiosis may drive this accelerated aging. First, we evaluated intestinal integrity, an indirect measure of microbial translocation, in PLWH on ART and controls by assessing the levels of tight junction proteins (ZO-1 and occludin) in the ileum and colon using immunofluorescence and a scaling method described in Supplementary Fig. 6. Data in Fig. 3A-B show that intestinal integrity, as assessed by levels of ZO-1 and occludin, was significantly lower in PLWH on ART compared to controls. This observation was also made among heterosexual individuals within each group, despite the small sample size (Supplementary Fig. 7A). This suggested that gut permeability was higher in PLWH on ART. Consistently, markers of gut damage and microbial translocation in plasma were higher in PLWH on ART compared to controls (Fig. 3C and Supplementary Fig. 7B). The damage/translocation markers assessed were REG3α (intestinal stress marker [74]), I-FABP (enterocyte apoptosis marker [75]), Zonulin (tight junction permeability marker [76, 77]), LPS binding protein (bacterial translocation marker [78]), β-glucan (fungal translocation marker [79]), and sCD163 (microbe-triggered myeloid inflammation marker). Together, these data suggest that in PLWH the intestinal integrity is compromised, resulting in enhanced microbial translocation.

Next, we investigated the relationships between the degree of intestinal integrity or microbial translocation and the two measures of biological aging. Specifically, we determined correlations between intestinal integrity (based on levels of tight junction proteins) or microbial translocation (based on levels of the damage/translocation markers) and either accelerated aging (calculated as in Fig. 1C-F using data from the epigenetic aging clocks in blood and tissues) or blood-based inflammatory aging markers (measured as in Fig. 2B). Correlation heat-maps (Fig. 3D and Supplementary Table 4) showed that intestinal integrity negatively (significantly, albeit weakly) correlated with accelerated biological aging and levels of inflammatory aging markers, while microbial translocation positively correlated with accelerated biological aging and levels of inflammatory aging markers. Moreover, the higher levels of HIV DNA and RNA in blood and/or tissues (as surrogates of HIV persistence) correlated with lower intestinal integrity (Fig. 3E-H). These findings highlight the connections between elevated intestinal permeability and microbial translocation, accelerated aging, greater inflammation, and greater HIV persistence in the blood and intestinal tissues of PLWH on ART.

### Living with HIV is linked to intestinal and fecal microbial dysbiosis, notably a decrease in butyrate-producing bacteria

As we described in the preceding sections, PLWH on ART have compromised intestinal integrity which may lead to accelerated biological aging both systemically and in tissues. One plausible mechanism underlying this compromised intestinal integrity is microbial dysbiosis. Microbial dysbiosis can pave the way for an increase in bacteria that produce toxic metabolites, such as those involved in tryptophan catabolism [27, 80, 81]. It can also cause a decline in bacteria that generate metabolites considered beneficial, such as short-chain fatty acids (SCFAs) [82], notably butyrate, which are microbiota-derived metabolites known to bolster intestinal barrier integrity [83]. With this context in mind, we probed the microbiome in stool, ileum, and colon samples from PLWH on ART and controls using 16S rRNA sequencing.

**Fig. 4:**
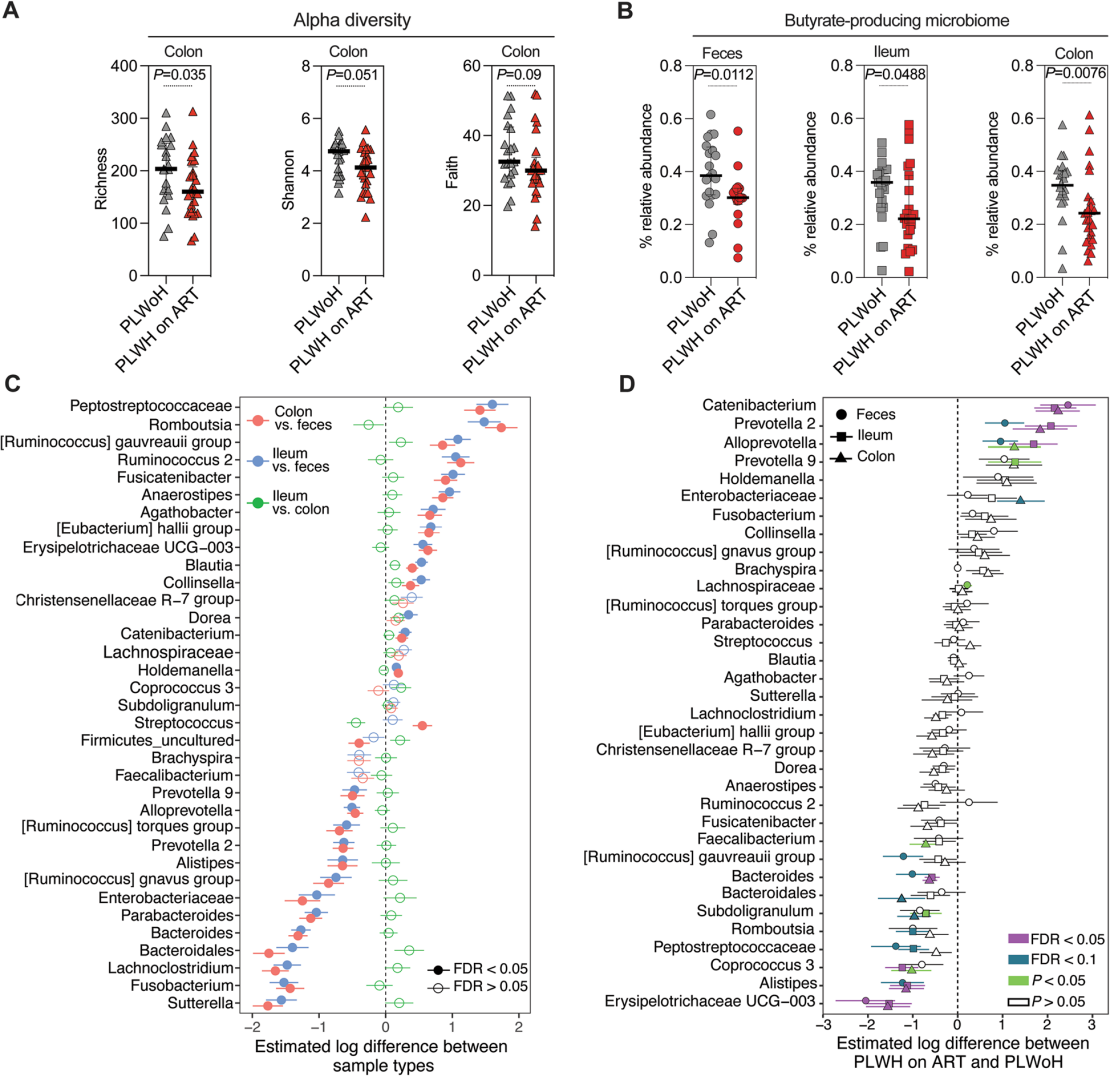
Living with HIV is associated with distinct intestinal and fecal microbial dysbiosis, characterized by a reduction in butyrate-producing bacteria. **A** Alpha diversity indices (Richness, Shannon, Faith’s phylogenetic diversity) reveal reduced colon microbiome diversity in PLWH on ART vs. controls. **B** Butyrate-producing microbiota's relative abundance in feces, ileum, and colon for both study groups are illustrated. Medians and IQR are depicted, with significance derived from Mann–Whitney U tests. **C** Differential bacterial abundance between tissue and fecal samples on a logarithmic scale. Comparisons include colon vs. feces (red), ileum vs. feces (blue), and ileum vs. colon (green). Significance markers: FDR < 0.05 (closed circles) and FDR > 0.05 (open circles). Adjustments made for multiple tests using the Benjamini–Hochberg method. **D** Log-scale differences in bacterial abundance across colon (triangle), ileum (square), and feces (circle) between PLWoH and PLWH on ART. Analysis incorporated bacterial taxa with > 1% mean relative abundance. Linear models estimated abundance changes, and adjustments for multiple tests used the Benjamini–Hochberg method. Significance markers: FDR < 0.05 (purple), FDR < 0.1 (cyan), and *P* < 0.05 (green)

We found that microbial alpha diversity, a hallmark of a healthy microbiota [84] as measured by various models (Richness, Shannon, and Faith), was lower in the colon of PLWH on ART compared to controls (Fig. 4A). This difference was mainly observed for Richness, although it should be noted that this metric is an inherently biased estimate of diversity. Smaller non-significant differences were observed in feces and ileum between the groups (Supplementary Fig. 8A-B). We also observed differences in beta-diversity between the two groups and between sample types (Supplementary Table 5 and Supplementary Fig. 9). We then assessed the relative abundance of bacteria known to produce SCFA, particularly butyrate, and the relative abundance of bacteria considered pro-inflammatory (“pathobionts”; Supplementary Table 6). The relative abundance of butyrate-producing bacteria was lower in PLWH on ART than in controls (Fig. 4B). PLWH on ART also tended to have a more pro-inflammatory fecal microbiome and lesser SCFA-producing fecal bacteria, but trends were not statistically significant (Supplementary Fig. 8C-D).

When we examined specific bacterial genera in the feces, colon, and ileum, we found that the microbiome in these locations varied significantly (Fig. 4C; FDR < 0.05). Comparing PLWH on ART with PLWoH (Fig. 4D), we found that living with HIV on ART was associated with an enrichment of some bacterial genera and a depletion of others, in feces, colon, and/or ileum. Enriched bacterial genera include putatively pro-inflammatory bacterial genera [23] such as *Catenibacterium*, *Prevotella* 2, *Alloprevotella*, *Prevotella* 9, and *Enterobacteriaceae.* Depleted genera included putatively anti-inflammatory bacteria [85, 86] and bacteria known for their ability to produce SCFAs such as *Erysipelotrichaceae* UCG − 003, *Alistipes*, *Coprococcus 3*, *Peptostreptococcaceae, Romboutsia*, *Subdoligranulum*, *Bacteroidales*, [Ruminococcus] *gauvreauii* group, and *Faecalibacterium*.

Similar to our analysis for the rates of acceleration of biological aging, we also conducted an additional analysis focusing on heterosexual participants within the two groups to explore whether the observed microbial dysbiosis is exclusively linked to sexual practices. Our findings, detailed in Supplementary Fig. 10, reveal that heterosexual PLWH on ART exhibit: 1) a trend towards a decrease in several indices of alpha diversity in feces or tissues (Supplementary Fig. 10A-C); 2) a decrease in levels of butyrate-producing bacteria in feces (but not in the colon and ileum tissues) (Supplementary Fig. 10D); and 3) differences in levels of several bacterial genera compared to their heterosexual PLWoH controls. However, most were not significant after multiple testing correction (FDR < 0.05), likely due to the small sample size relative to the entire cohort (Supplementary Fig. 10E). This reinforces findings from earlier studies [27, 82], suggesting an HIV-related microbial imbalance. This microbial imbalance may contribute to the previously observed decrease in intestinal integrity and, consequently, the accelerated biological aging in PLWH on ART.

### A distinct mucosal microbial signature is linked to accelerated biological aging

**Fig. 5:**
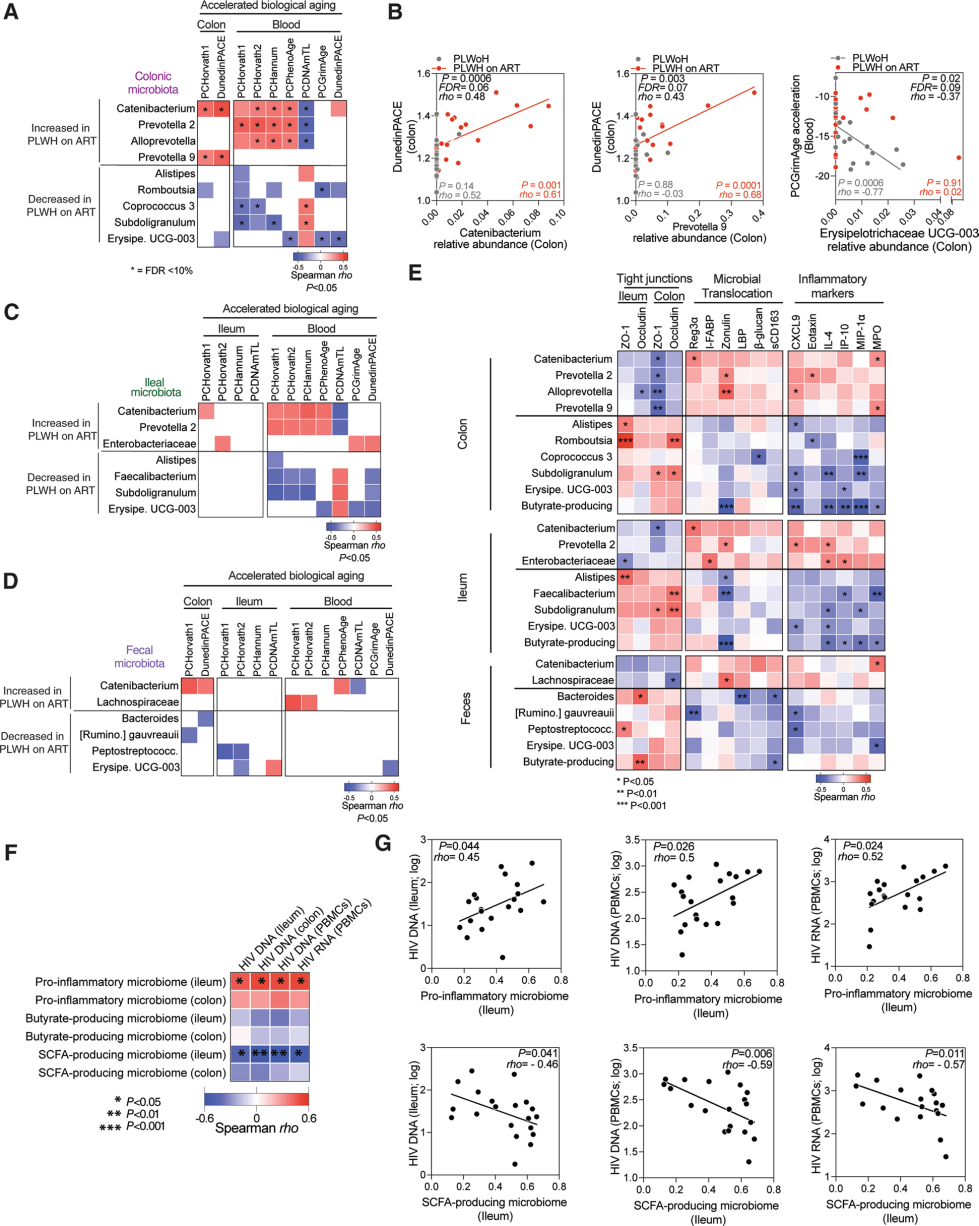
Microbial signature in mucosa linked to accelerated biological aging. **A** Spearman’s rank correlation analysis depicts associations between colonic microbiomes enriched (top rows) or depleted (bottom rows) in PLWH on ART and accelerated biological aging in the colon and blood (columns). Red and blue signify correlations with *P* < 0.05. White indicates *P* > 0.05. *FDR < 10%.** B** Plots represent correlations between specific bacterial taxa in the colon and accelerated epigenetic aging: *Catenibacterium* vs. DunedinPACE (colon) [left]; *Prevotella 9* vs. DunedinPACE (colon) [middle]; and *Erysipelotrichaceae* UCG-003 vs. GrimAge (blood) [right]. **C-D** Spearman’s rank correlation heatmaps display association of ileal microbiota (**C**) and fecal microbiota (**D**) with accelerated aging in different regions. Red indicates positive and blue indicates negative correlations with *P* < 0.05; white spaces show *P* > 0.05. **E** Spearman’s rank correlation heatmap illustrates correlations between microbiomes in the colon (top), ileum (middle), and feces (bottom) with tight junction integrity, microbial translocation, and inflammatory markers. **F** Spearman’s rank correlation heatmap depicts associations between pro-inflammatory, butyrate, and SCFA-producing microbiota in the ileum or colon with HIV DNA/RNA levels. Red signifies positive and blue indicates negative correlations. * *P* < 0.05, ** *P* < 0.01, *** *P* < 0.001. **G** Spearman’s rank correlations are demonstrated between the relative abundance of pro-inflammatory microbiome (ileum) vs. HIV DNA/RNA in ileum and PBMCs (top). The bottom panel shows correlations of SCFA-producing microbiome (ileum) vs. HIV DNA/RNA in ileum and PBMCs

Given the dysbiosis observed in PLWH (Fig. 4D), we next asked if this dysbiosis was related to the accelerated biological aging we had observed in PLWH. Our analyses in Fig. 5A and Supplementary Table 7 revealed that specific bacterial genera that were enriched in colon tissue from PLWH on ART (such as *Catenibacterium*, *Prevotella 2*, *Alloprevotella*, and *Prevotella 9*) correlated strongly with greater accelerated aging (FDR < 10%). In contrast, other genera that were depleted in colon tissue from PLWH on ART (like *Erysipelotrichaceae UCG-003*, *Alistipes*, *Coprococcus 3*, *Romboutsia*, and *Subdoligranulum*) correlated with slower accelerated aging. Notably, the correlations between the enriched bacteria and higher accelerated biological aging were driven by samples from PLWH on ART, whereas the correlations between the depleted bacteria and slower accelerated biological aging were driven by samples from PLWoH (Fig. 5B). Similar analyses using ileal (Fig. 5C and Supplementary Table 7), and fecal (Fig. 5D and Supplementary Table 7) samples did not yield any correlations with FDR < 10%, although some nominal P values were significant.

Beyond their associations with accelerated biological aging rates, taxa enriched in PLWH on ART were linked to lower tight junction protein levels in tissues, elevated microbial translocation, and enhanced inflammation (Fig. 5E**,** top rows of each section, and Supplementary Table 8). In contrast, taxa that were depleted in PLWH on ART were associated with better intestinal integrity, lower microbial translocation, and lower inflammation (Fig. 5E**,** bottom rows of each section, and Supplementary Table 8). Separate analyses revealed that the pro-inflammatory microbiome was associated with higher levels of HIV DNA and RNA in both blood and tissues. By contrast, the SCFA-producing bacteria, notably those producing butyrate, associated with lower levels of HIV DNA and RNA (Fig. 5F-G). These findings suggest that certain bacterial genera, especially those from the colon, may influence the pace of biological aging. Moreover, they shed light on the intricate relationship between microbial profiles, inflammation, HIV persistence, and the biological aging trajectory in PLWH on ART.

### Correlation networks reveal links between the mucosal microbiota, microbe-related metabolites, and accelerated biological aging

Building on our observations (Fig. 5) that SCFAs were associated with slower biological aging, we expanded our inquiry to other microbe-related metabolites. Recognizing that many effects of the microbiota are mediated by metabolites other than SCFAs, we conducted an untargeted metabolomic analysis on stool and plasma samples from both PLWH on ART and controls. Our goal was to identify additional metabolites that might bridge the microbial signature (Fig. 5) with the accelerated biological aging patterns observed.

**Fig. 6:**
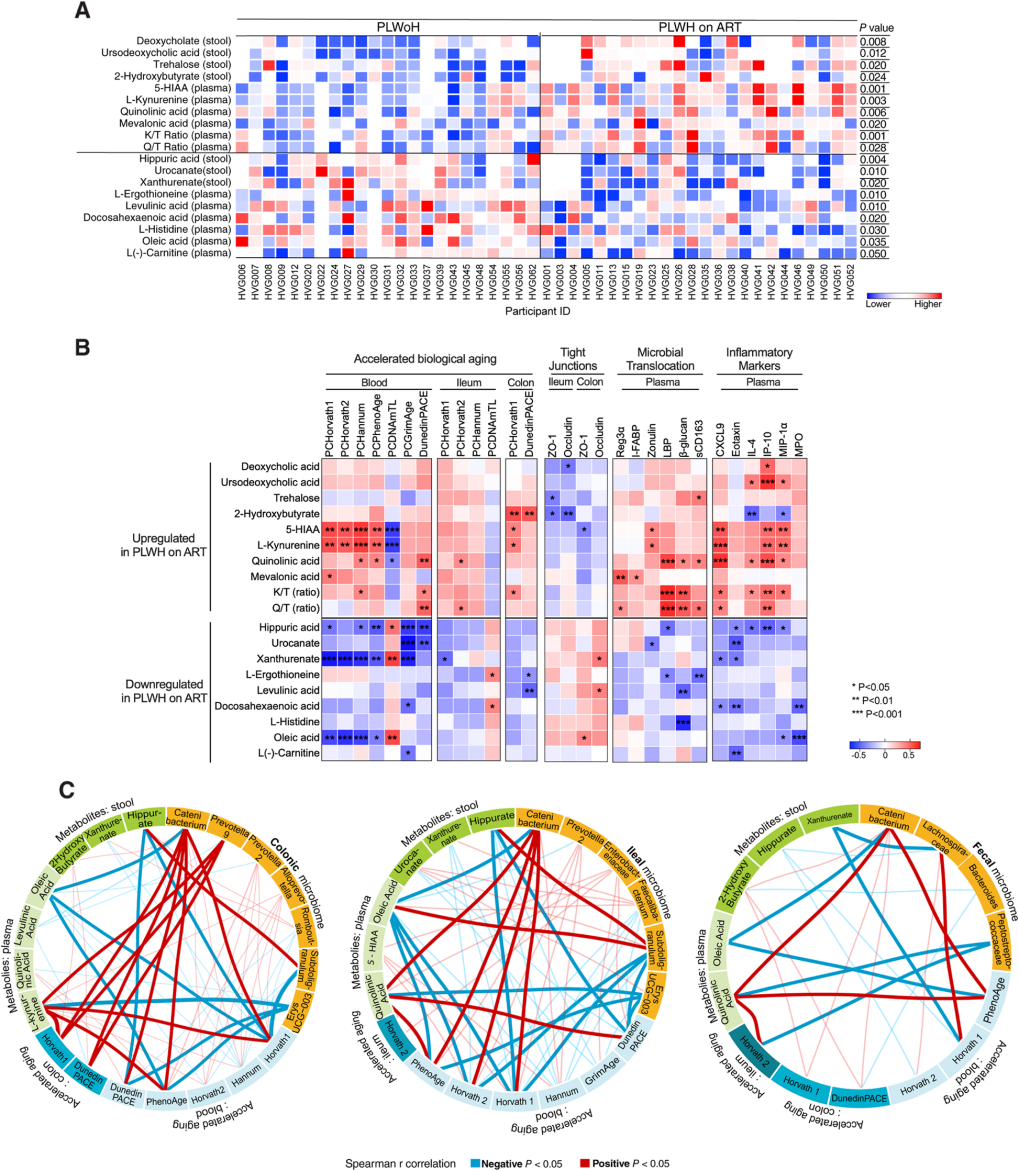
Correlation network reveals specific links between the mucosal microbiome, microbiota-associated metabolites, and accelerated biological aging. **A** A heatmap displays the relative abundance of metabolites associated with gut health in both stool and plasma samples from PLWH on ART and PLWoH. The color gradient, from blue to red, signifies the normalized metabolite values, with red representing higher abundance and blue indicating lower abundance. Differences between groups were assessed using the Mann–Whitney U test. **B** Spearman’s rank correlation heatmap demonstrates relationships between gut-specific metabolites (rows) and indicators of accelerated biological aging, tight junction integrity, microbial translocation, and inflammatory markers (columns). The top panel highlights metabolites upregulated in PLWH on ART, while the bottom section presents metabolites that are downregulated in this group. Positive and negative correlations are represented by red and blue, respectively. Key: * *P* < 0.05, ** *P* < 0.01, *** *P* < 0.001. **C** Circos plots visualize Spearman’s rank correlations among tissue-specific microbiome (yellow), metabolites derived from plasma and stool samples (green), and indicators of accelerated biological aging specific to both tissue and blood (blue). Red lines denote significant positive correlations, while blue lines signify negative correlations. Only correlations manifesting a 3-way association are included. Emphasized lines hint at their potential functional relevance. 5-HIAA stands for 5-Hydroxyindole-3-acetic acid, K/T ratio stands for Kynurenine/Tryptophan ratio, and Q/T ratio stands for Quinolinic acid/Tryptophan ratio

First, we assessed a spectrum of microbiota- and gut-specific metabolites (Supplementary Table 9). PLWH on ART had elevated levels of metabolites known to be detrimental, such as L-kynurenine and quinolinic acid, both by-products of tryptophan catabolism [80, 87]. We confirmed this by evaluating two common measures of tryptophan catabolism, the kynurenine to tryptophan (K/T) ratio and the quinolinic acid to tryptophan (Q/T) ratio [88]. Both ratios were indeed higher in PLWH on ART than controls (Fig. 6A). Further, PLWH on ART had lower levels of metabolites associated with microbial diversity and intestinal health, like hippuric acid [89], L-ergothioneine [90], and oleic acid [91] (Fig. 6A). This metabolic imbalance was also observed in an analysis focusing on heterosexual participants, suggesting that it is not solely dependent on sexual practices (Supplementary Fig. 11). The metabolites enriched in PLWH on ART were associated with accelerated biological aging, compromised intestinal integrity, heightened microbial translocation, and greater inflammation (Fig. 6B Supplementary Table 10). Conversely, metabolites that were less abundant in PLWH on ART correlated with slower biological aging, greater intestinal integrity, lower microbial translocation, and reduced inflammation (Fig. 6B and Supplementary Table 10).

To visualize these complex interactions, we performed a network analysis, which illustrated distinct three-way interactions among microbial genera enriched in PLWH on ART, elevated tryptophan catabolism metabolites, diminished beneficial gut metabolites like hippuric acid and oleic acid, and accelerated biological aging (Fig. 6C). Conversely, the network analysis also identified distinct connections among microbial genera depleted in PLWH on ART, diminished tryptophan catabolism metabolites, abundant protective gut metabolites, and slower biological aging (Fig. 6C). These intricate relationships were most pronounced in the colon, followed by the ileum, and then the feces, underscoring the tissue-specific microbial imprints of accelerated biological aging which were absent in the fecal microbiome.

## Discussion

Previous research indicates that, even under ART, HIV infection accelerates biological aging in the blood [32, 35, 92]. Yet, the effects of HIV on biological aging in the intestines—a primary site for HIV persistence and pathogenesis—and the links between age and increased gut permeability, microbial translocation, and viral persistence are not known. In our study, we used a systems biology approach to examine colon, ileum, and blood samples from PLWH on ART and PLWoH. Our findings reveal that living with HIV is associated with an acceleration or accentuation of biological aging in the ileum and colon at rates different from that of the blood. Importantly, we identified specific bacterial taxa and associated microbial metabolic signatures that are linked to both intestinal and systemic biological aging. These insights pave the way for further research into the mechanisms underlying these connections and potential strategies to prevent or delay aging-related complications in PLWH.

We identified specific microbial signatures linked to biological aging in mucosal tissues, but not in feces. A primary factor responsible for diminished intestinal integrity and microbial translocation is microbial dysbiosis, an imbalance in the intestinal microflora. A healthy gut microbiota strikes a delicate balance between beneficial commensals and pathobionts. However, living with HIV tilts this equilibrium, favoring the proliferation of pathobionts and opportunistic pathogens in the gut. Although many studies have focused on the fecal microbiome, the bacteria in mucosal biopsy samples (termed the mucosal-associated microbiome) can differ significantly from those in feces [23, 93]. Furthermore, HIV-related shifts in the mucosal microbiome aren't always mirrored in fecal samples from the same individual [23]. Our findings highlight a pronounced difference between the mucosal-associated and fecal-associated microbiomes, with only the former closely tied to accelerated biological aging. This underscores the importance of examining the microbiome across different anatomical sites. Recognizing the variations among microbiomes and their links to diverse biological conditions, like biological aging, can lead to specialized strategies. Such tactics could counter microbial dysbiosis, strengthen intestinal integrity, and prevent both intestinal and systemic inflammation, thereby slowing the accelerated biological aging process.

Specific bacterial genera, including *Catenibacterium*, *Prevotellaceae*, and *Enterobacteriaceae*, enriched in PLWH on ART, were strongly associated with accelerated biological aging. These bacterial taxa can catabolize tryptophan [27] and were correlated with elevated levels of the metabolic byproducts of tryptophan catabolism. Increased tryptophan catabolism leads to an accumulation of toxic metabolic byproducts such as kynurenine and quinolinic acid. These byproducts have been linked with adverse outcomes in chronic HIV infection [27, 80, 81, 87, 88]. For example, quinolinic acid, a known neurotoxin and activator of the *N*-methyl-D-aspartate (NMDA) receptor, has been connected to neurological complications in HIV infection [94, 95]. Likewise, elevated kynurenine levels are associated with neurological deficits in the aging population [96, 97]. Our study consistently identified strong correlations among bacterial taxa capable of inducing tryptophan catabolism, the generation of these toxic metabolic byproducts, and the acceleration of biological aging at both the systemic and intestinal levels. However, more research is needed to determine the specific bacterial species initiating tryptophan catabolism and to understand if these associations with biological aging are causative. Identifying such causal relationships could set the stage for creating therapeutic strategies to counteract the rapid onset of biological aging. For instance, inhibitors of IDO-1 (indoleamine 2,3-dioxygenase 1, the rate-limiting enzyme involved in tryptophan catabolism), like Epacadostat and Linrodostat, have been tested in cancer trials (often in tandem with immune checkpoint inhibitors) to block tryptophan depletion, and might offer promising intervention routes [27].

Conversely, certain bacterial taxa, including *Erysipelotrichaceae* UCG-003, *Coprococcus*, *Faecalibacterium*, and *Subdoligranulum*, were significantly lower in PLWH and were associated with slower rates of biological aging. Some of these bacterial species, like *Erysipelotrichaceae* UCG-003, have been previously associated with healthy aging [98], although their potential role in promoting healthy aging in PLWH was not established prior to our study. Many of these bacteria are known for their ability to produce SCFAs, which are essential for maintaining gut health. Among SCFAs, butyrate is a primary energy source for intestinal epithelial cells and plays a pivotal role in modulating T cell responses in the gut [99]. This metabolite, recognized as an HDAC inhibitor (HDACi), has strong anti-inflammatory properties, which helps to maintain the intestinal barrier's integrity [83]. Consistently, our study revealed a marked reduction in butyrate-producing bacterial genera, such as *Coprococcus*, and *Subdoligranulum*, in both the ileum and colon; *Faecalibacterium* in colon; and [Ruminococcus] *gauvreauii* group in fecal samples from PLWH on ART compared to controls. Moreover, these bacteria were positively associated with the maintenance of tight junction integrity and negatively associated with markers of inflammation, microbial translocation, and the acceleration of biological aging. These findings underscore the potential role of SCFA production in maintaining intestinal barrier integrity and fostering the intestines' healthy aging. Such insights provide a foundation for investigating strategies to enhance SCFA levels, like the adoption of SCFA-promoting prebiotics, to potentially slow the aging process in the intestinal environment.

In addition to SCFAs, we identified strong associations between other microbiota-related metabolites — which possess well-established anti-inflammatory properties — and a decelerated rate of both intestinal and systemic aging. Notable among these are hippuric acid, L-ergothioneine, and oleic acid. Hippurate, produced through microbial activity in the colon, is often used as a marker for good gut health and increased microbial diversity [89]. Similarly, L-ergothioneine serves as an indicator of a healthy gut microbiota and has shown antioxidant properties, helping counteract oxidative stress in intestinal contexts, as observed in vitro and in animal models [100, 101]. Oleic acid, with its anti-inflammatory properties, has been found to boost alpha diversity in older individuals with HIV when supplemented [91]. Taken together, these insights indicate that living with HIV may induce changes in the gut microbiota, leading to disruptions in key, modifiable, microbiota-related metabolic pathways. Such disruptions might contribute to weakened intestinal integrity, increased microbial translocation, and ongoing inflammation. These factors could potentially contribute significantly to the process of both local and systemic biological aging.

Various confounding factors, including sexual orientation and practices, can influence the intestinal microbiota composition. For instance, *Catenibacterium* and *Prevotella* are more prevalent in men who have sex with men (MSM), regardless of their HIV status [24]. Yet, microbial dysbiosis also occurs in PLWH regardless of their sexual orientation or practices [30]. In our study, a significant portion of PLWH identified as MSM. Therefore, the potential influence of sexual practices on their gut microbiota cannot be disregarded. Nevertheless, our study included several analyses focused on heterosexual participants within both PLWoH and PLWH on ART. In these analyses, we observed differences in rates of acceleration in biological aging, intestinal permeability, microbial translocation, microbiome composition, and metabolites between the two groups. This suggests that sexual practices are not the sole driver of these differences. The differences and correlations we observed may be driven by a combination of factors, including living with HIV on ART, sexual practices, as well as demographic and non-demographic factors associated with HIV. Future research should carefully examine the distinct impacts of HIV, specific ART regimens, sex/gender, sexual practices, and other potential confounding variables on the relationship between the intestinal microbiome and both intestinal and systemic biological aging. Such studies will require large and diverse participant cohorts, as well as controlled animal studies, to gain a comprehensive understanding of these intricate interactions. Nevertheless, our study reveals novel associations between intestinal integrity, microbial translocation, microbial dysbiosis, and biological aging that may be applicable to a wide range of conditions and factors associated with a high incidence of aging-associated diseases.

Our study has several limitations, including: 1) While our human-based study cannot unequivocally demonstrate mechanistic links between the enrichment of pro-inflammatory microbial taxa, the depletion of anti-inflammatory microbial taxa, and the acceleration of biological aging, existing literature on these bacterial taxa aligns with and supports our findings and hypotheses. Nevertheless, detailed mechanistic insights will require further research using intestinal organoids and animal models. 2) The human gut microbiota is not composed of bacteria alone; it encompasses fungi, archaea, protists, and viruses, each vital for both intestinal and systemic health. Recent studies emphasize the role of protists like *Blastocystis* in metabolizing tryptophan, affecting immune activation and CD4^+^ T cell responses [102]. Also, β-glucan (a marker of fungal translocation) is associated with systemic inflammation in PLWH on ART, and PLWH have an altered gut virome with enriched eukaryotic viruses linked to gastrointestinal diseases [103]. As such, the impact of this diverse microflora on aging merits deeper investigation. 3) We have pinpointed associations between bacterial taxa, microbial metabolites, accelerated aging, and levels of cell-associated HIV DNA in both intestines and blood. These findings hint at intricate interactions between the gut microbiota, its metabolic activities, aging, and HIV persistence. However, it's crucial to note that the majority of cell-associated HIV DNA in PLWH on ART is defective [104, 105]. Future work should investigate links between a pro-aging microbiome, intact HIV reservoirs in the intestines, and the relationship between active HIV persistence and faster aging. This may reveal a feedback loop where increased HIV persistence exacerbates immune dysfunction, necessitating further research.

## Conclusions

Effective ART has revolutionized HIV therapeutics over the past decade and significantly increased the lifespan of PLWH. However, this longevity has been accompanied by a high incidence of several age-related non-AIDS associated co-morbidities such as cardiovascular and renal diseases, neurocognitive impairments, and ailments of the gut. Our study unveils previously unrecognized links between specific intestinal microbial signatures, their metabolic activity, and accelerated biological aging. These insights pave the way for novel interventions targeting the microbiota and metabolites, aiming to strengthen the intestinal barrier, decelerate aging, and reduce inflammation-associated diseases in PLWH and other population with chronic inflammation stemming from a compromised intestinal barrier.

### Supplementary Information

**Additional file 1:** **Supplementary Fig. 1. **Evaluation of biological age in gut tissues and blood.

**Additional** **file 2:** **Supplementary Fig. 2. **Rate of acceleration of biological aging in blood and tissues among heterosexual PLWoH and PLWH on ART**.**

**Additional file 3:** **Supplementary Fig. 3.** Rate of biological aging acceleration among PLWH on different ART regimens.

**Additional file 4:** **Supplementary Fig. 4. **Assessment of telomere lengths in PBMCs.

**Additional file 5:** **Supplementary Fig. 5.** Correlation analysis of HIV DNA levels and biological age.

**Additional file 6:** **Supplementary Fig. 6. **Visualization of tight junction integrity scores.

**Additional file 7:** **Supplementary Fig. 7. **Higher intestinal permeability and microbial translocation in heterosexual PLWH on ART compared to heterosexual PLWoH.

**Additional file 8:** **Supplementary Fig. 8. **Microbiome alpha diversity and relative abundance.

**Additional file 9:** **Supplementary Fig. 9. **Microbiome beta diversity.

**Additional file 10:** **Supplementary Fig. 10. **Microbiome dysbiosis in heterosexual PLWH on ART.

**Additional file 11:** **Supplementary Fig. 11. **Differential levels of plasma and stool metabolites between heterosexual PLWoH and heterosexual PLWH on ART.

**Additional file 12:** **Supplementary Table 1.** Read counts of the microbiome data.

**Additional file 13:** **Supplementary Table 2.** Spearman's rank correlations between telomere lengths (blood) and DNA methylation-based biological aging (related to Figure 2A).

**Additional file 14:** **Supplementary Table 3. **Spearman's rank correlations between accelerated biological aging and Inflammatory markers (plasma) - related to Figure 2C.

**Additional file 15:** **Supplementary Table 4. **Spearman's rank correlations between accelerated biological aging, tight junction proteins, markers of microbial translocation, and inflammation markers (related to Figure 3D).

**Additional file 16:** **Supplementary Table 5. **Beta-diversity between PLWoH and PLWH on ART.

**Additional file 17:** **Supplementary Table 6. **Lists of a) SCFA producing bacteria; b) butyrate producing bacteria; and c) pro-inflammatory bacteria.

**Additional file 18:** **Supplementary Table 7. **Spearman's rank correlations between the microbiome and accelerated biological aging (related to Figure 5A, C, D).

**Additional file 19:** **Supplementary Table 8. **Spearman's rank correlations between accelerated biological aging, tight junction proteins, markers of microbial translocation, and inflammation markers (related to Figure 5E).

**Additional file 20:** **Supplementary Table 9. **A list of gut-related metabolites.

**Additional file 21:** **Supplementary Table 10. **Spearman's rank correlations between metabolites and accelerated biological aging, tight junction proteins, markers of microbial translocation, and inflammation markers (related to Figure 6B).

## Data Availability

Raw microbiome data are available at the Sequence Read Archive (SRA) dataset ProjectID PRJNA1030421 (https://www.ncbi.nlm.nih.gov/bioproject/PRJNA1030421). Raw Epigenetic DNA methylation data are available at Gene Expression Omnibus (GEO): GSE245924 (https://www.ncbi.nlm.nih.gov/geo/query/acc.cgi?acc=GSE245924).

## Abbreviations

PLWH: People living with HIV
ART: Antiretroviral therapy
SCFA: Short-chain fatty acid
BMI: Body mass index
PNA: Peptide Nucleic Acid
OCT: Optimal cutting temperature
QC: Quality Control
LC–MS: Liquid chromatography-mass spectrometry
HILIC: Hydrophilic interaction liquid chromatography
IDA: Information dependent acquisition
PBMC: Peripheral blood mononuclear cells
TL: Telomere lengths
HT-Q-FISH: High-throughput quantitative fluorescence in situ hybridization
iAGE: inflammatory aging
LBP: LPS Binding Protein
Q/T: Quinolinic acid/Tryptophan
K/T: Kynurenine/Tryptophan
IQR: Interquartile range
5-HIAA: 5-Hydroxyindole-3-acetic acid
NMDA: N-methyl-D-aspartate
IDO-1: Indoleamine 2,3-dioxygenase 1
HDACi: HDAC inhibitor
MSM: Men who have sex with men
TERT: Telomerase Reverse Transcriptase
TAVs: telomere-associated variables
MPO: Myeloperoxidase
