# Supplementary figures and images for: Distinct intestinal microbial signatures linked to accelerated systemic and intestinal biological aging
Source: Microbiome. 2024 Feb 22;12:31. doi: 10.1186/s40168-024-01758-4 (PMC10882811; doi:10.1186/s40168-024-01758-4)

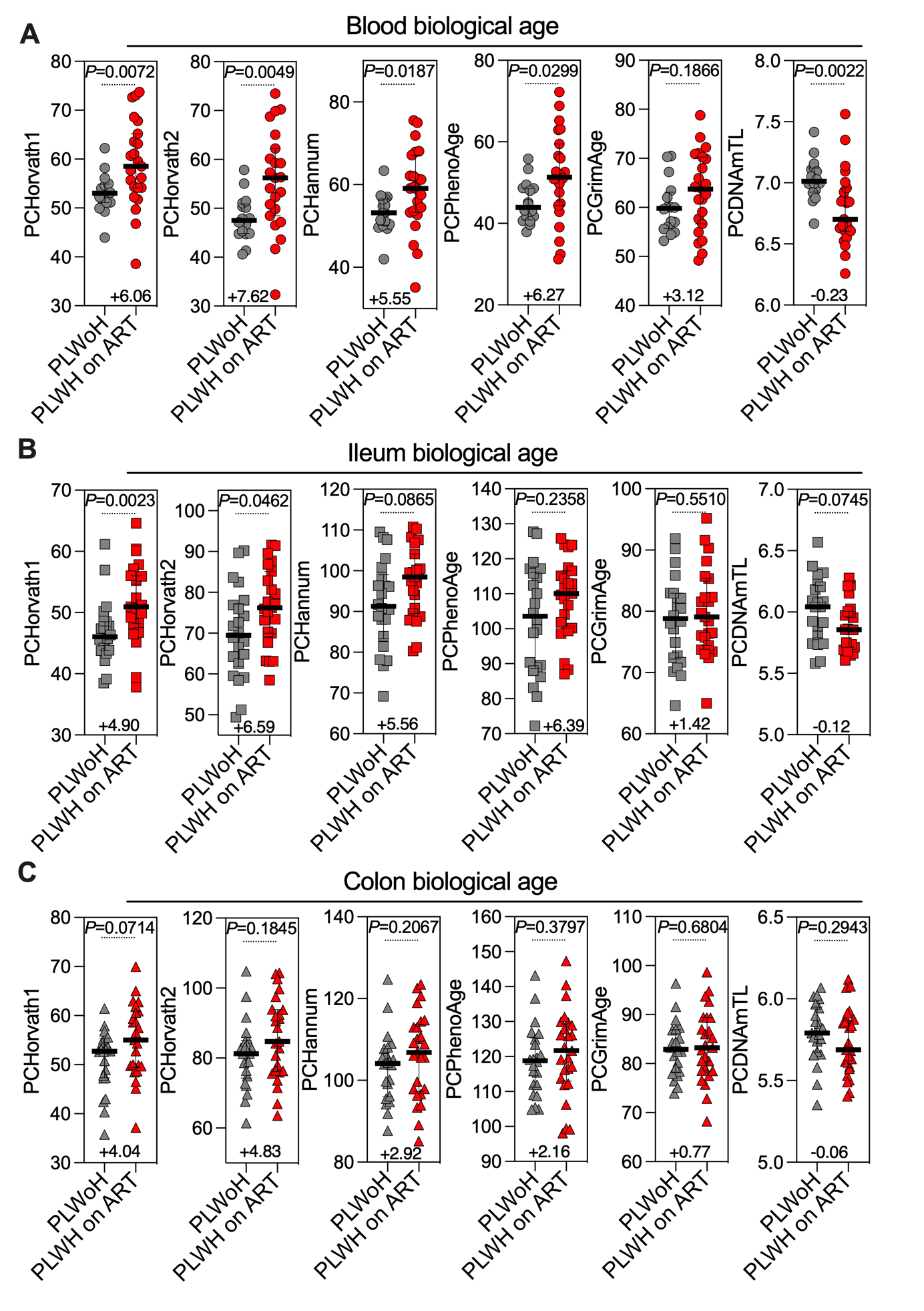

Supplement: Supplementary file 2 — Additional file 1: Supplementary Fig. 1. Evaluation of biological age in gut tissues and blood. [file 40168_2024_1758_MOESM1_ESM.tiff]

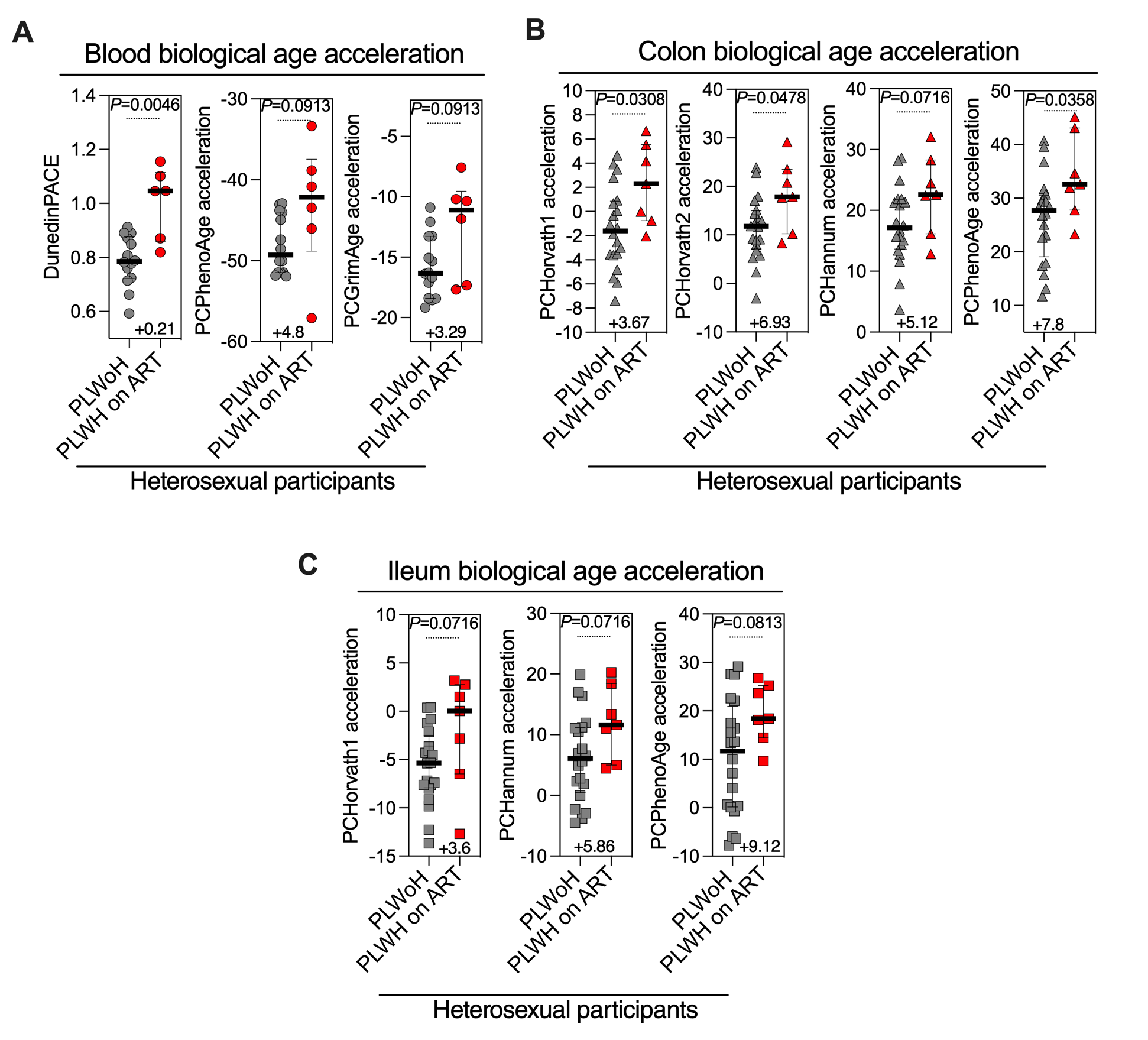

Supplement: Supplementary file 3 — Additional file 2: Supplementary Fig. 2. Rate of acceleration of biological aging in blood and tissues among heterosexual PLWoH and PLWH on ART. [file 40168_2024_1758_MOESM2_ESM.tiff]

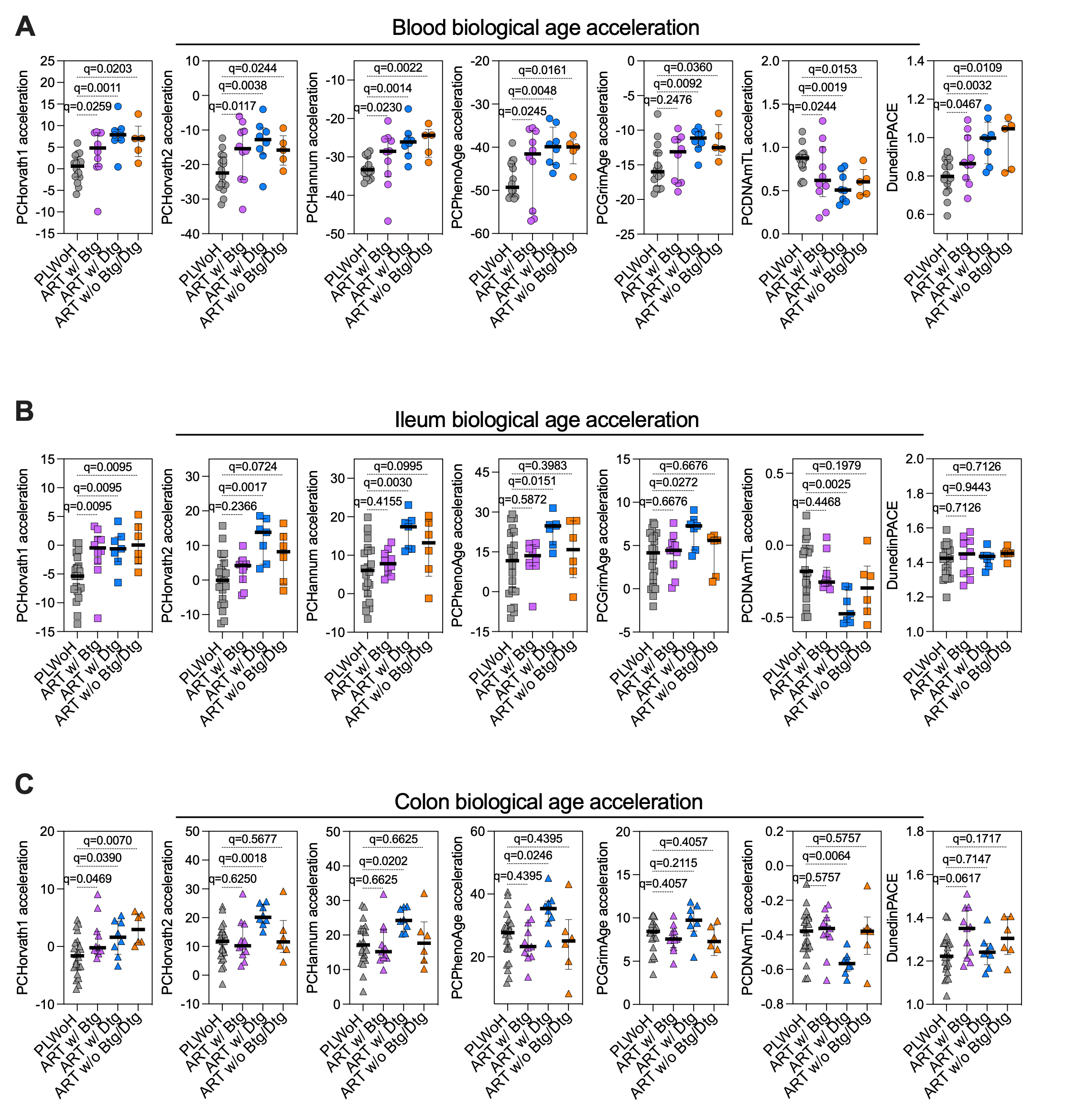

Supplement: Supplementary file 4 — Additional file 3: Supplementary Fig. 3. Rate of biological aging acceleration among PLWH on different ART regimens. [file 40168_2024_1758_MOESM3_ESM.tiff]

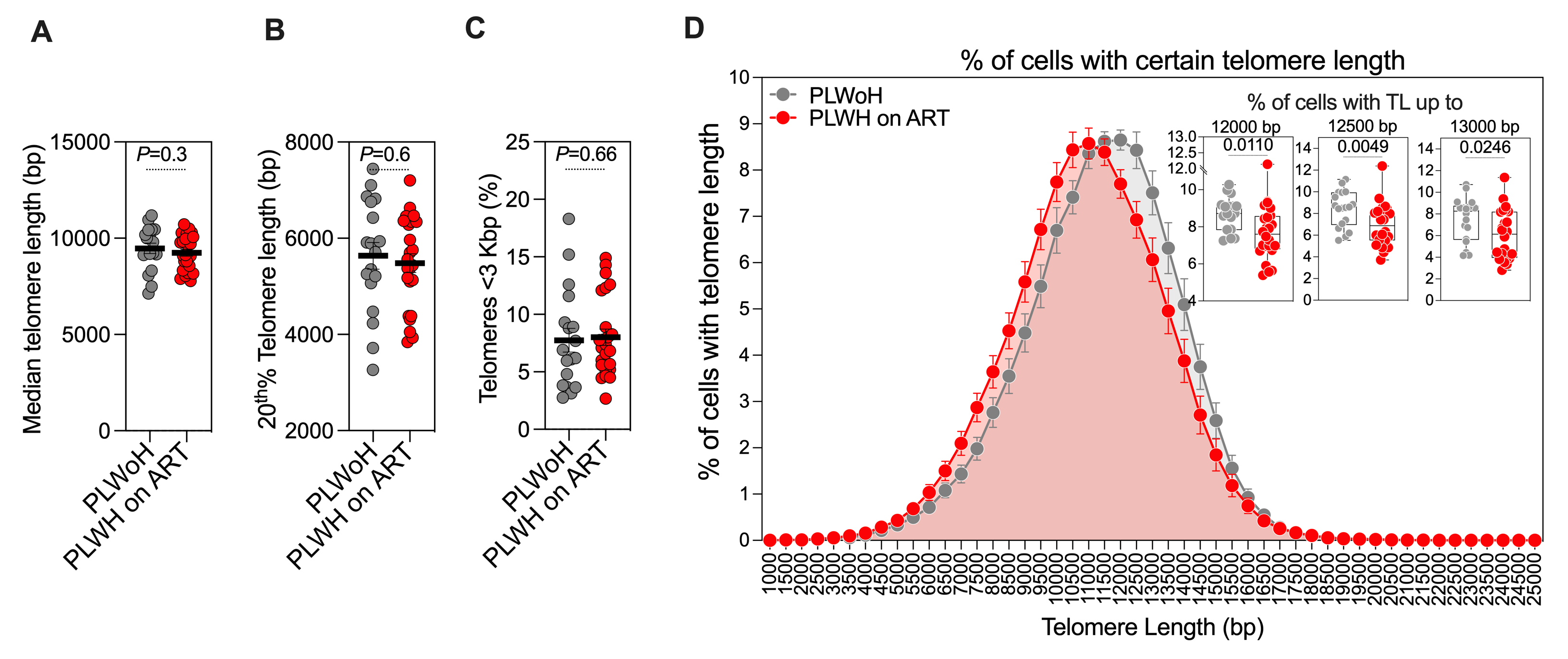

Supplement: Supplementary file 5 — Additional file 4: Supplementary Fig. 4. Assessment of telomere lengths in PBMCs. [file 40168_2024_1758_MOESM4_ESM.tiff]

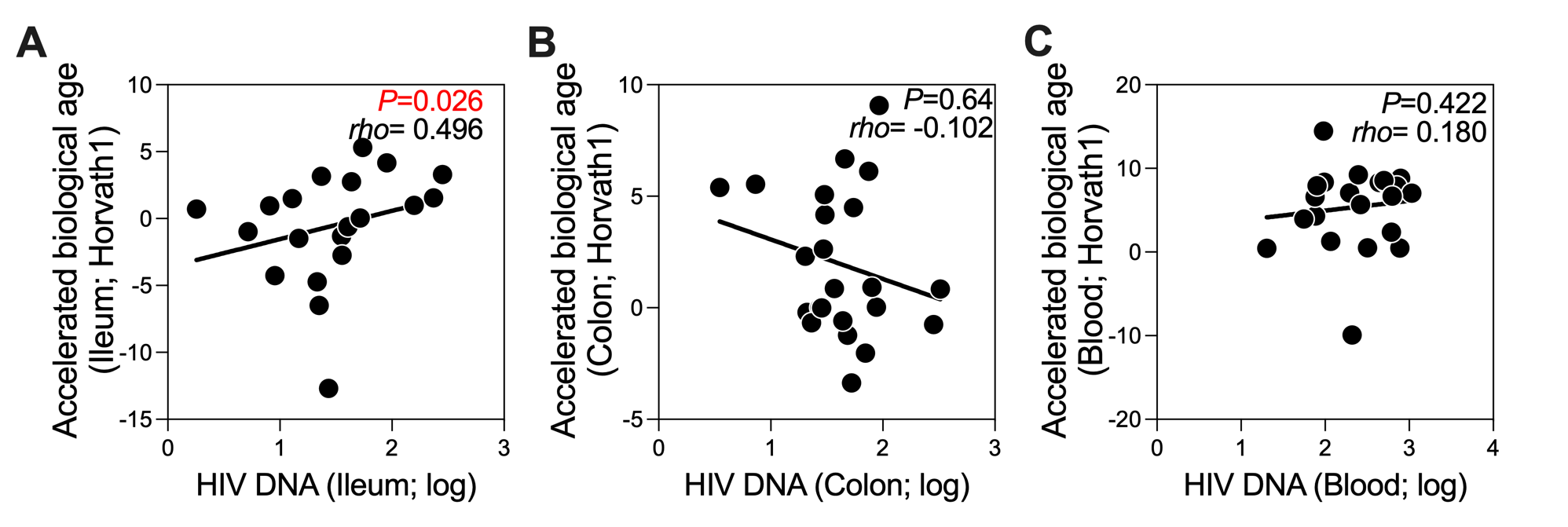

Supplement: Supplementary file 6 — Additional file 5: Supplementary Fig. 5. Correlation analysis of HIV DNA levels and biological age. [file 40168_2024_1758_MOESM5_ESM.tiff]

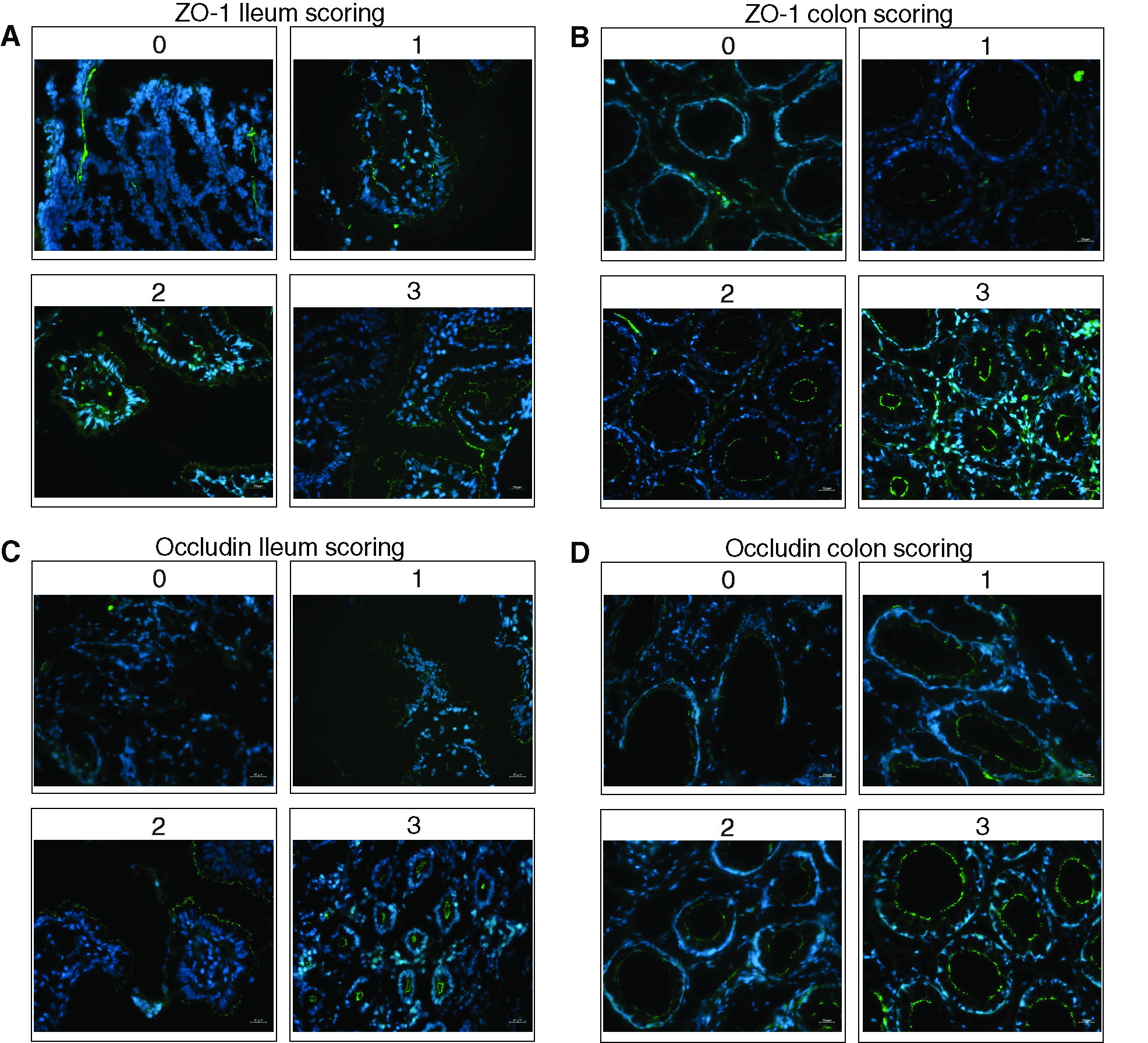

Supplement: Supplementary file 7 — Additional file 6: Supplementary Fig. 6. Visualization of tight junction integrity scores. [file 40168_2024_1758_MOESM6_ESM.tif]

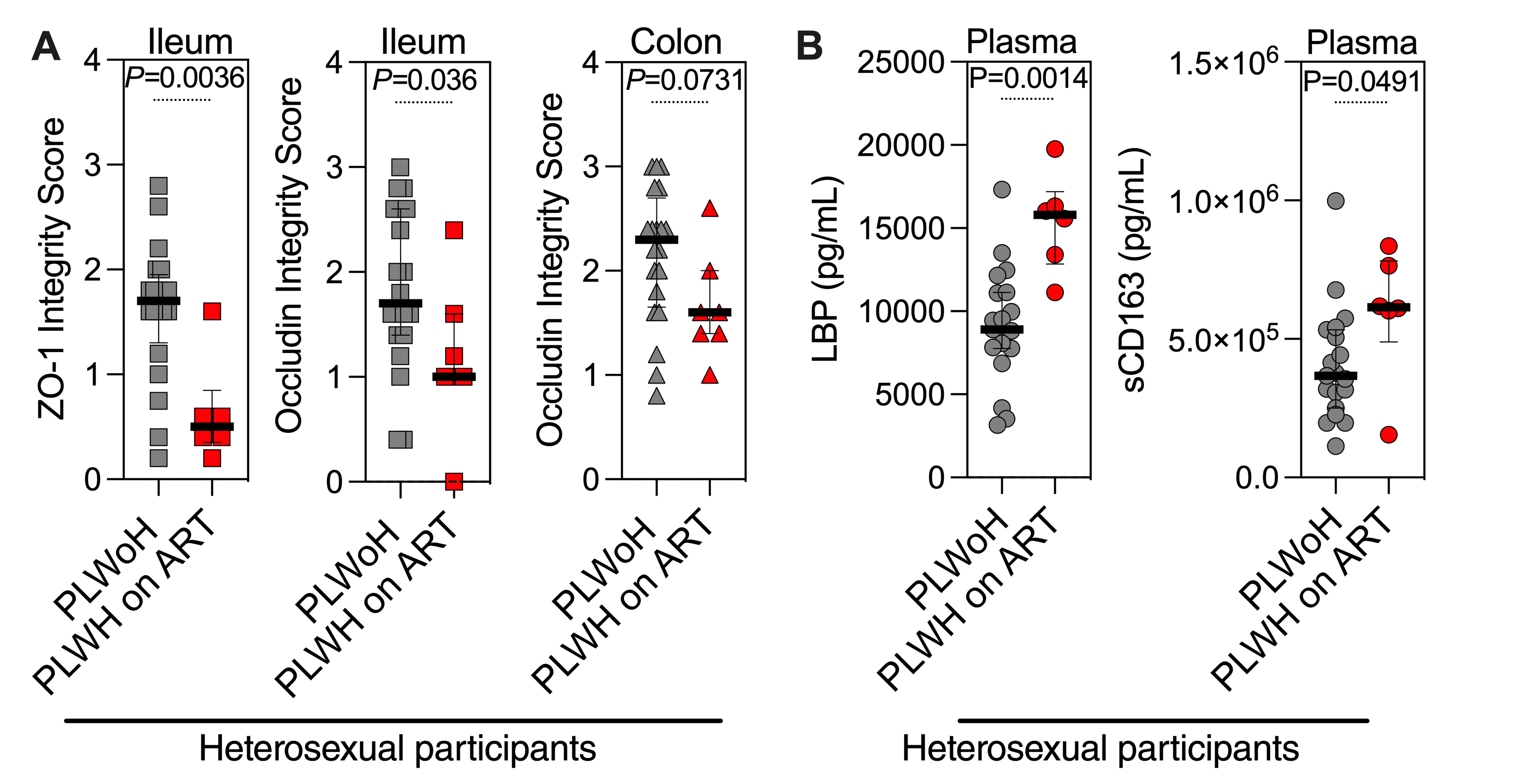

Supplement: Supplementary file 8 — Additional file 7: Supplementary Fig. 7. Higher intestinal permeability and microbial translocation in heterosexual PLWH on ART compared to heterosexual PLWoH. [file 40168_2024_1758_MOESM7_ESM.tiff]

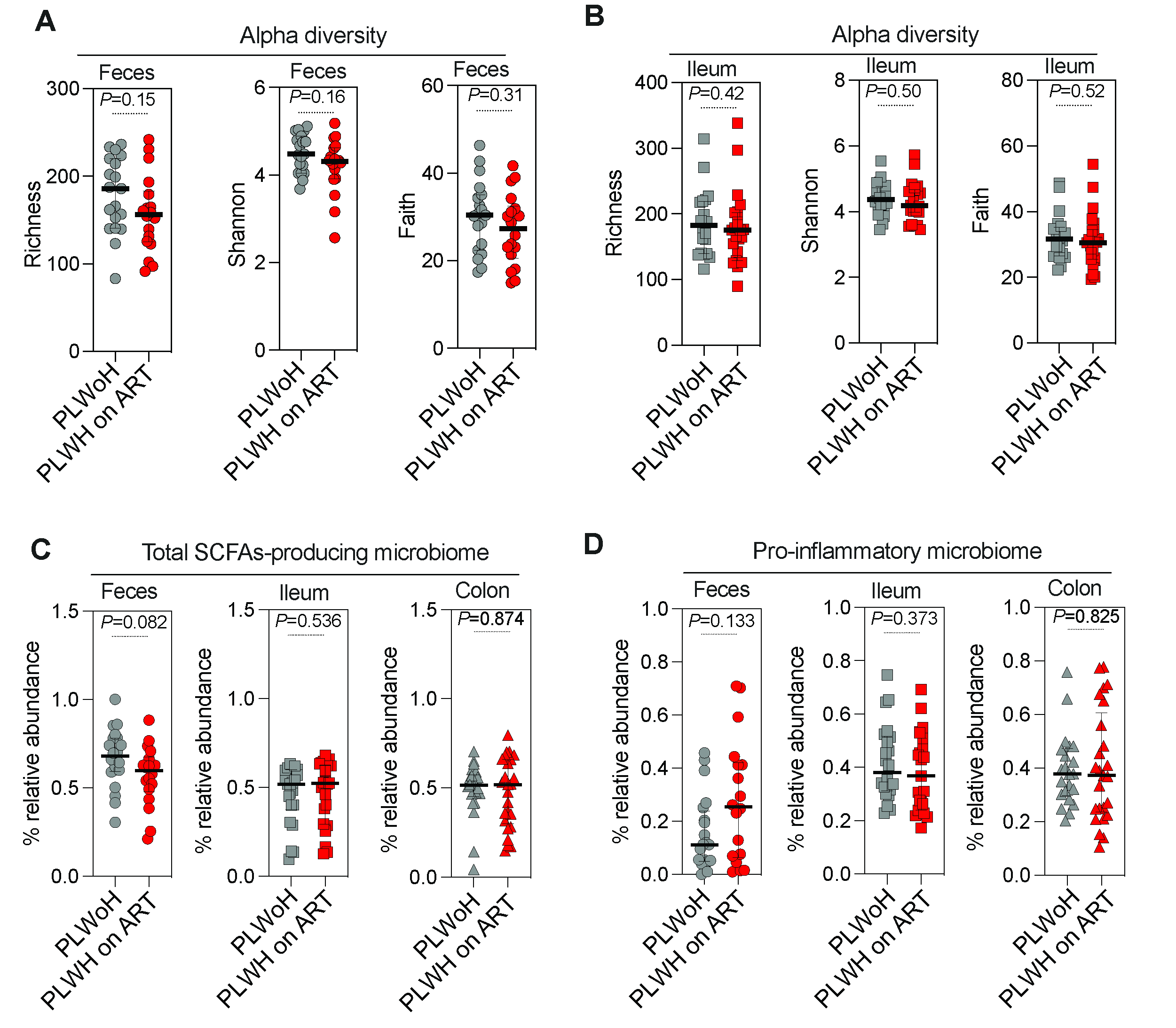

Supplement: Supplementary file 9 — Additional file 8: Supplementary Fig. 8. Microbiome alpha diversity and relative abundance. [file 40168_2024_1758_MOESM8_ESM.tif]

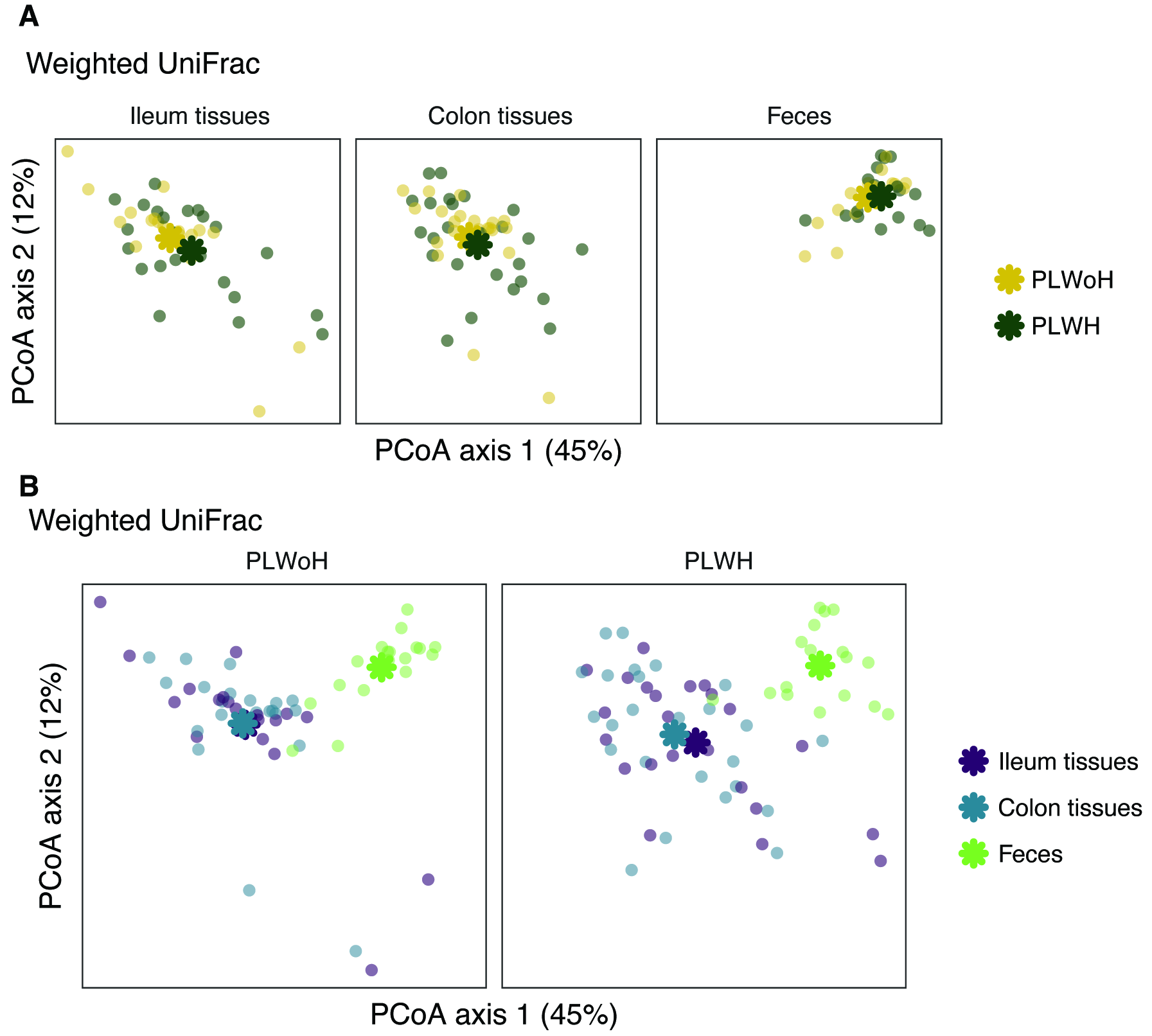

Supplement: Supplementary file 10 — Additional file 9: Supplementary Fig. 9. Microbiome beta diversity. [file 40168_2024_1758_MOESM9_ESM.tif]

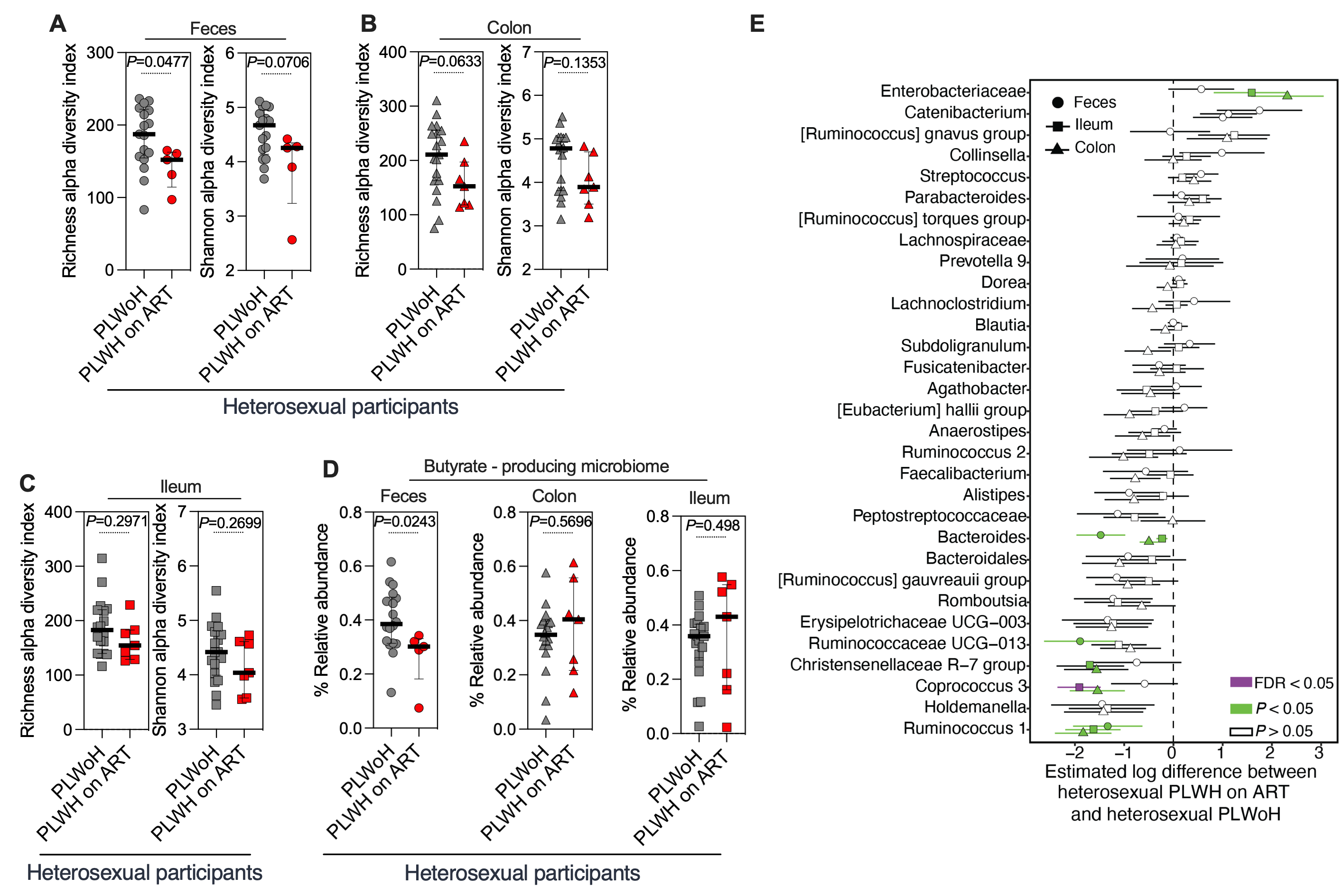

Supplement: Supplementary file 11 — Additional file 10: Supplementary Fig. 10. Microbiome dysbiosis in heterosexual PLWH on ART. [file 40168_2024_1758_MOESM10_ESM.tiff]

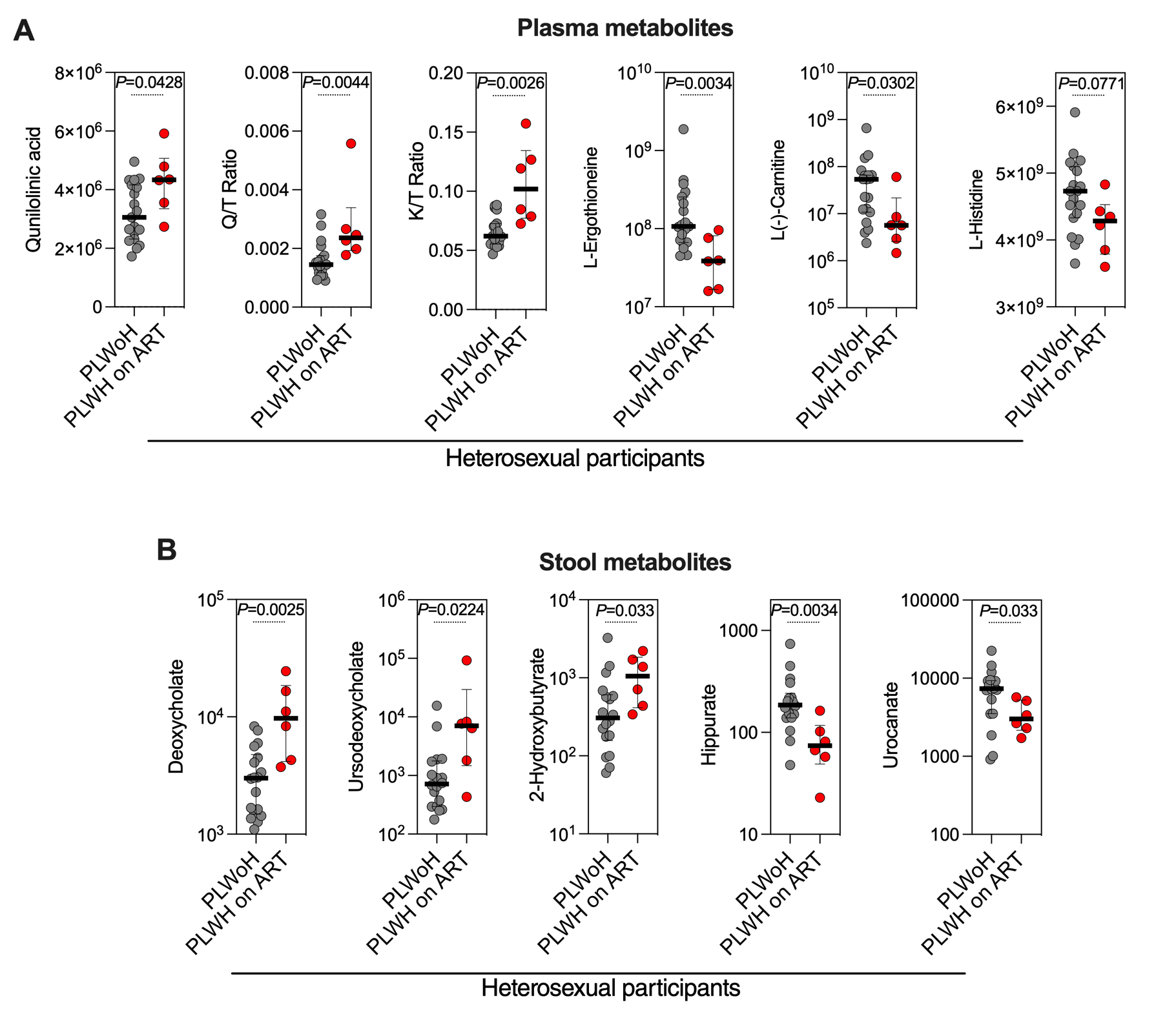

Supplement: Supplementary file 12 — Additional file 11: Supplementary Fig. 11. Differential levels of plasma and stool metabolites between heterosexual PLWoH and heterosexual PLWH on ART. [file 40168_2024_1758_MOESM11_ESM.tiff]
